# β_2_-adrenergic receptor promotes liver regeneration partially through crosstalk with c-met

**DOI:** 10.1038/s41419-022-04998-0

**Published:** 2022-06-27

**Authors:** Xiang Tao, Can Chen, Yingxiang Chen, Luoying Zhang, Jiong Hu, Hongjun Yu, Minglu Liang, Qin Fu, Kai Huang

**Affiliations:** 1 Department of Cardiology, Union Hospital, Tongji Medical College, Huazhong University of Science and Technology, Wuhan, 430000 China; 2 Clinical Center of Human Gene Research, Union Hospital, Tongji Medical College, Huazhong University of Science and Technology, Wuhan, 430000 China; 3 Department of Pharmacology, School of Basic Medicine, Tongji Medical College, Huazhong University of Science and Technology, Wuhan, China; 4 Key Laboratory of Molecular Biophysics of the Ministry of Education, College of Life Science and Technology, Huazhong University of Science and Technology, Wuhan, China; 5 Department of Histology and Embryology, School of Basic Medicine, Tongji Medical College, Huazhong University of Science and Technology, Wuhan, China; 6 Department of Biochemistry and Molecular Biology, School of Basic Medicine, Tongji Medical College, Huazhong University of Science and Technology, Wuhan, China

**Keywords:** Cell proliferation, Endocytosis

## Abstract

The β_2_-adrenergic receptor (β_2_AR) is a G protein-coupled receptor (GPCR) that mediates the majority of cellular responses to external stimuli. Aberrant expression of β_2_AR results in various pathophysiological disorders, including tumorigenesis, but little is known about its role in liver regeneration. This study aims to investigate the impact and the underlying mechanism of β_2_AR in liver regeneration. Here, we found that β_2_AR was upregulated during liver regeneration induced by 70% PH. Deletion of β_2_AR in mice resulted in 62% mortality 2 days post-PH, decreased proliferative marker expression and impaired liver function throughout regeneration. Moreover, AAV8-mediated overexpression of β_2_AR in hepatocytes accelerated the regeneration process and increased target gene expression. Mechanistically, β_2_AR recruited G-protein-coupled receptor kinase 2 (GRK2) to the membrane and then formed a complex with c-met to transactivate c-met signaling, which triggered downstream extracellular regulated protein kinase (ERK) signaling activation and nuclear translocation. Inhibition of c-met with SU11274 or ERK with U0126 decreased β_2_AR overexpression-induced hepatocyte proliferation. Our findings revealed that β_2_AR might act as a critical mediator regulating liver regeneration by crosstalk with c-met and activation of ERK signaling.

## Introduction

The liver has considerable regenerative capacity [1]. In response to partial hepatectomy (PH) or acute injury, quiescent hepatocytes can be triggered to re-enter the cell cycle and divide to restore tissue homeostasis [2–4]. Understanding the mechanisms during liver regeneration is crucial to promote regeneration and prevent clinical complications during liver transplantation. Previous studies have shown that a large number of genes are involved in liver regeneration, but the essential circuitry required for the process may involve the participation of cytokines, growth factors and metabolic networks [1]. However, the molecular regulatory mechanisms of liver regeneration are still elusive.

Three types of transmembrane receptors transmit extracellular mitotic signals, including ion channel-linked receptors, enzyme-linked receptors, and G protein-coupled receptors (GPCRs). The roles of enzyme-linked receptors (including growth factor receptors and cytokine receptors) and ion channel-linked receptor cascades involved in liver regeneration have been studied extensively [5–8]. However, little is known about the role of GPCRs in the regenerating liver.

β_2_-Adrenergic receptors (β_2_ARs) are prototypical GPCRs mediating diverse cellular signals, often in a ligand-specific manner [9]. Several studies in mice and humans reinforce the growing recognition of the role of β_2_AR in cancer progression, metastasis, and drug resistance in various tissues [10–12]. Other evidence has demonstrated that β_2_AR activation can promote cell proliferation to improve functional recovery from injuries in skeletal muscle, vasculature and heart [13–15]. Moreover, β_2_AR is closely involved in the regulation of cytokines, growth factors and metabolic networks that are essential for liver regeneration. Although the levels of β_2_AR are elevated in hepatocytes from regenerating livers [16], the detailed mechanisms remain unclear. In the present study, we used a β_2_AR knockout mouse model and adeno-associated virus 8 (AAV8)-mediated overexpression of β_2_AR in mouse liver to define the biological function of β_2_AR in liver regeneration post-PH.

## Results

### β_2_AR was upregulated during liver regeneration induced by 70% PH

**Fig. 1:**
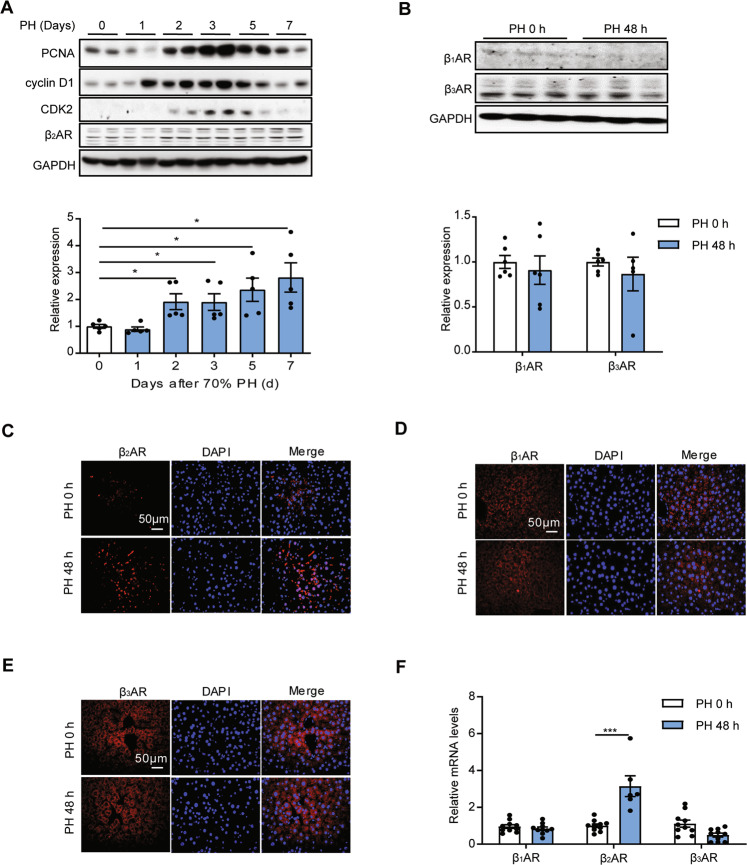
β_2_AR was upregulated during liver regeneration induced by 70% PH. **A** Protein expression levels of β_2_AR, PCNA, cyclin D1 and CDK2 in the liver at the indicated times post PH. **B** Protein expression levels of β_1_AR and β_3_AR 48 h post PH (*n* = 5–6). **C**–**E** Immunofluorescence staining of PH mouse livers for the indicated proteins. **F** The mRNA levels of βARs 48 h post-PH. Data are shown as means ± SEM. *P < 0.05, **P < 0.01, ***P < 0.001 by two-tailed Student’s t test.

Partial hepatectomy is regarded as a classic model that has been widely used in the experimental analysis of the molecular mechanisms underlying liver regeneration [3]. To investigate the role of β_2_AR in liver regeneration, we determined changes in the expression of β_2_AR in C57BL/6J mice over a time course post-PH using Western blotting. Concomitant with increased expression of proliferation markers such as proliferating cell nuclear antigen (PCNA), cyclin D1, cyclin-dependent kinase 2 (CDK2), β_2_AR protein levels started increasing at 48 h post-PH (Fig. 1A). Nevertheless, the protein levels of β_1_AR and β_3_AR were not changed by PH (Fig. 1B). Additionally, β_2_AR was upregulated post-PH by immunofluorescence staining, while β_1_AR and β_3_AR levels were not changed (Fig. 1C, E). Consistently, the mRNA expression of β_2_AR but not β_1_AR and β_3_AR was increased at 48 h post PH (Fig. 1F). Thus, these findings indicated that β_2_AR was upregulated during liver regeneration after 70% PH in mice.

### β_2_AR translocated to the nucleus during liver regeneration induced by 70% PH

**Fig. 2:**
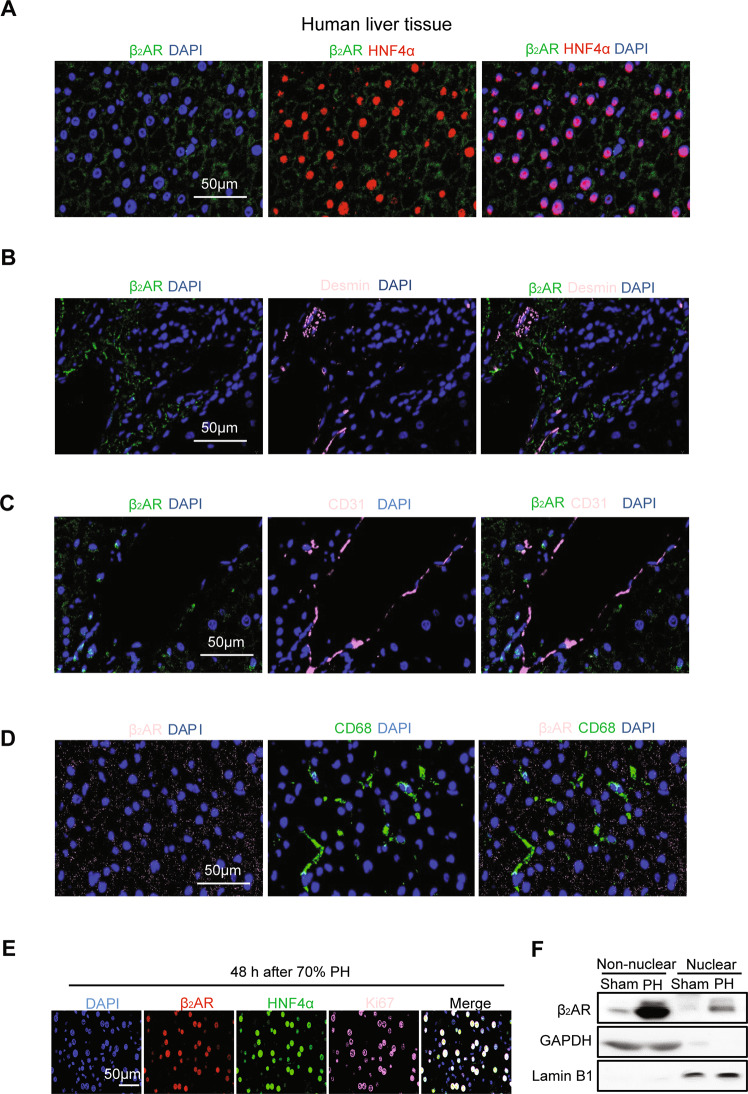
β_2_AR translocated to the nucleus during liver regeneration induced by 70% PH. **A**–**D** Immunofluorescence staining of β_2_AR and DAPI in human liver with the indicated cell markers HNF4α (hepatocytes), desmin (hepatic stellate cells), CD31 (sinusoidal endothelial cells) and CD68 (Kupffer cells). **E** Immunofluorescence staining of β_2_AR, Ki67, HNF4α and DAPI in mouse livers 48 h post PH. **F** The nuclear and non-nuclear components of liver tissues from Sham and PH groups were isolated and the expression of β_2_AR were detected by western blot.

β_2_AR is a member of the 7-transmembrane family of receptors. Intracellular signaling after β_2_AR activation is largely affected through cyclic adenosine monophosphate and protein kinase A [17]. To clarify the precise distribution of β_2_AR in liver tissues, we analyzed the subcellular localization of β_2_AR in normal human tissues by fluorescence immunohistochemistry. Notably, β_2_AR displayed a membrane location in mature hepatocytes marked by hepatocyte nuclear factor 4α (HNF4α) (Fig. 2A). In addition, β_2_AR showed little colocalization with stellate cells, liver sinusoidal endothelial cells and Kupffer cells, which were marked by desmin, CD31 and CD68, respectively (Fig. 2B–D). Most remarkably, 48 h after PH, β_2_AR colocalized with Ki67 in the nucleus, indicating that it was translocated to the nucleus in proliferating mouse liver cells (Fig. 2E). To further validate the migration of β_2_AR, nuclear and non-nuclear components of liver tissues from Sham and PH groups were isolated and the expression of β_2_AR was detected by western blot (Fig. 2F). Thus, these findings indicated that β_2_AR was translocated to the nucleus during liver regeneration induced by 70% PH.

### β_2_AR deficiency impaired liver regeneration following 70% PH challenge

**Fig. 3:**
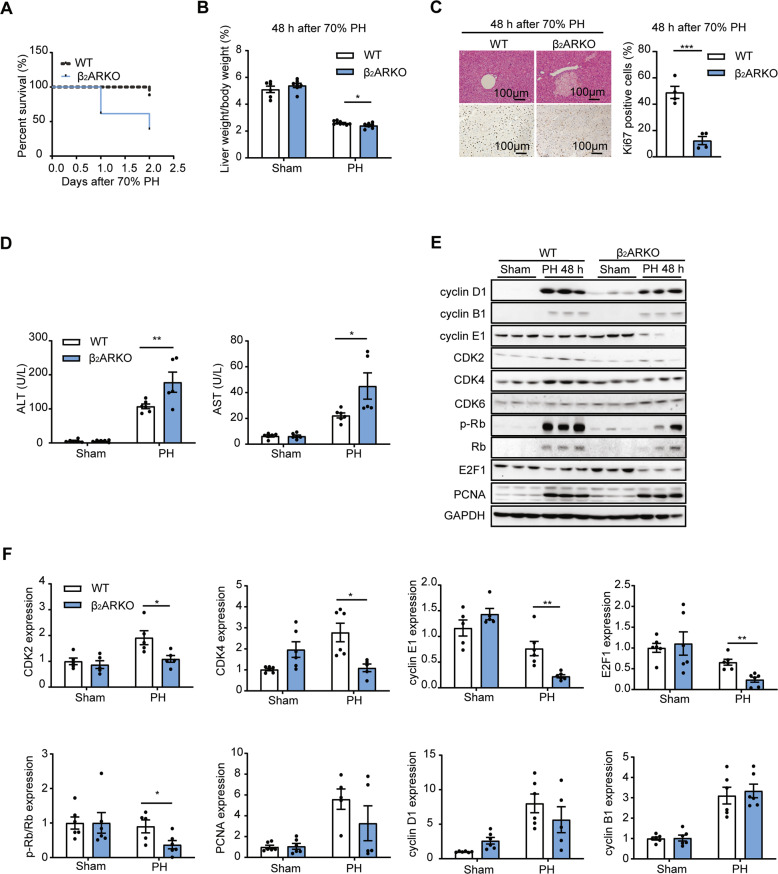
β_2_AR deficiency impaired liver regeneration following 70% PH challenge. β_2_ARKO and WT mice were challenged with 70% PH, and the remaining livers were collected at the indicated times. The survival curve after PH of β_2_ARKO and WT mice (**A**) and liver weight to body weight ratio (*n* = 6–9, **B**). **C** HE staining (upper panel) and Ki67 (lower panel) staining of β_2_ARKO and WT mouse livers 48 h post PH. The percentage of Ki67-positive cells was measured (*n* = 4). **D** The levels of ALT and AST in the serum of β_2_ARKO and WT mice 48 h post PH. **E** The protein levels of cell cycle markers in β_2_ARKO and WT mouse livers 48 h post PH (*n* = 5–6). **F** Quantitative analysis of immunoblotting. Data are shown as means ± SEM. *P < 0.05, **P < 0.01, ***P < 0.001 by two-tailed Student’s t test.

To investigate the role of β_2_AR in liver regeneration, we performed 70% PH on wild-type (WT) and β_2_AR knockout (β_2_ARKO) mice. To our surprise, approximately 62% of the β_2_ARKO mice died within 48 h after surgery, and the surviving mice exhibited much slower liver recovery rates. The hepatic index (ratio between liver and body weight) was found to be slightly decreased in β_2_ARKO mice (Fig. 3A, B). As indicated by hepatic tissue section staining, local necrosis was observed in β_2_ARKO mouse livers 48 h postoperation (Fig. 3C). Since transient steatosis is a metabolic hallmark of the early phase of liver regeneration [18], we performed oil red O staining and found that hepatic triglyceride levels were significantly decreased in β_2_ARKO mouse livers compared with WT mice (Fig. S1A). Moreover, β_2_AR deficiency decreased hepatocyte proliferation as evidenced by decreased Ki67 staining (Fig. 3C). Compared with WT mice, β_2_ARKO mice showed more serious liver injury in response to 70% PH, as evidenced by a slight rise in plasma levels of alanine transaminase (ALT) and aspartate transaminase (AST) (Fig. 3D).

Next, we investigated the underlying mechanisms of the decreased liver regeneration phenotypes in β_2_ARKO mice. Given that activation of the cell cycle plays an important role in the stimulation of robust hepatocyte proliferation during liver regeneration [4], the expression levels of checkpoint components of the cell cycle machinery were measured. As shown in Fig. 3E, F, the expression levels of CDK4, which appeared in the early G1 phase, were low in the livers of β_2_ARKO mice. Furthermore, cyclin E1, which interacts with CDK2 to form a complex that promotes the G1/S transition [19–21], was dramatically lower in β_2_AR-deficient livers. Consistently, impaired Rb phosphorylation and subsequent E2F1 translocation to the nucleus were found in β_2_ARKO mouse livers (Figs. 3E, F and S1B). These results suggested that β_2_AR deficiency resulted in a decreased G1 to S transition. Thus, these findings indicated that β_2_AR deficiency impaired liver regeneration following 70% PH challenge.

### Adeno-associated virus-mediated overexpression of β_2_AR promoted liver regeneration post PH

**Fig. 4:**
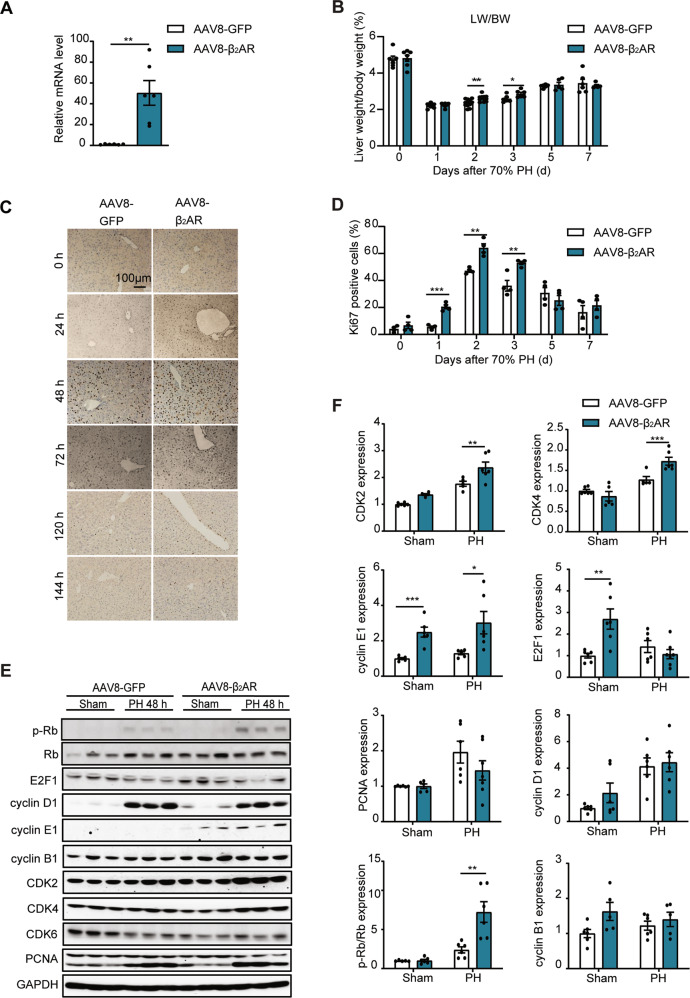
Adeno-associated virus-mediated overexpression of β_2_AR promoted liver regeneration post PH. **A** RT–qPCR analysis of the mRNA expression of β_2_AR (*n* = 6). **B** Liver weight to body weight ratio (*n* = 5–11). **C** Ki67 staining of mouse livers at the indicated times post PH (*n* = 4). **D** The percentage of Ki67-positive cells was measured (*n* = 4). **E** The protein levels of cell cycle markers in AAV8-GFP and AAV8-β_2_AR mouse livers 48 h post PH (*n* = 5–7). **F** Quantitative analysis of immunoblotting (*n* = 5–7). Data are means ± SEM. *P < 0.05, **P < 0.01, ***P < 0.001 with a two-tailed Student’s t test.

To further investigate the gain-of-function effects of β_2_AR on liver regeneration, adeno-associated virus 8 (AAV8)-mediated gene delivery in the liver was performed in mice. Four weeks later, we performed surgery on AAV8-GFP- and AAV8-β_2_AR-treated mice. As shown in Fig. 4A, the mRNA levels of β_2_AR in AAV8-β_2_AR-treated mouse livers were nearly 50 times higher than those in control GFP mice. Moreover, the hepatic index was slightly higher 48 and 72 h post PH after AAV8-β_2_AR treatment (Fig. 4B). Consistently, overexpression of β_2_AR resulted in increased hepatocyte proliferation at 24, 48, and 72 h post-PH (Fig. 4C, D). As shown in Fig. 4E, F, immunoblots showed that AAV8-β_2_AR treatment caused a profound increase in cell cycle genes in the liver of mice 48 h post PH, such as CDK2, CDK4, p-Rb/Rb and cyclin E1, while other genes were not significantly changed. Together, these results indicated that adeno-associated virus-mediated overexpression of β_2_AR promoted liver regeneration post PH.

### β_2_AR played an important role in primary hepatocyte proliferation

**Fig. 5:**
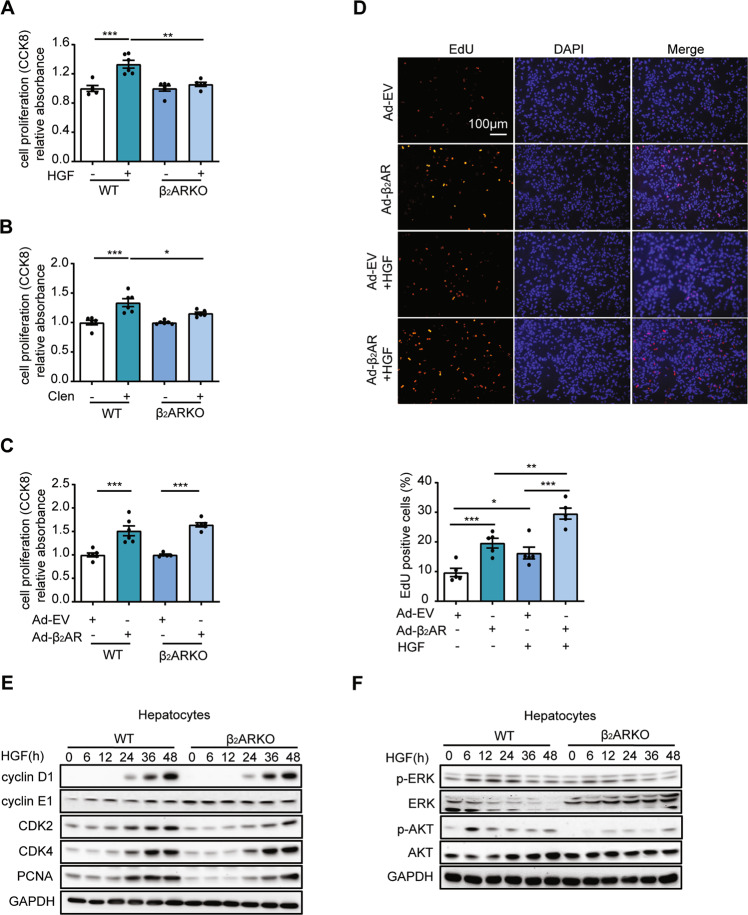
β_2_AR was essential for the regulation of hepatocyte proliferation. **A** Primary hepatocytes from WT and β_2_ARKO mice were treated with HGF (40 ng/ml) for 24 h, and then a CCK-8 assay was performed. **B** Primary hepatocytes from WT and β_2_ARKO mice were treated with Clen (clenbuterol, 10^−6^ mol/L) for 24 h, and then a CCK-8 assay was performed. **C** Primary hepatocytes from WT and β_2_ARKO mice were treated with Ad-EV or Ad-β_2_AR for 24 h, and then a CCK-8 assay was performed. **D** EdU staining was performed in Ad-β_2_AR- and Ad-EV-treated hepatocytes in the presence or absence of HGF. The percentage of EdU-positive cells was analyzed. **E** Primary hepatocytes from WT and β_2_ARKO mice were treated with HGF for the indicated times. Western blot analysis of cell cycle markers was performed. **F** Western blot analysis of p-ERK/ERK and p-AKT/AKT. Data are shown as means ± SEM. *P < 0.05, **P < 0.01, ***P < 0.001 by one-way ANOVA and post hoc Tukey’s test.

To further explore the role of β_2_AR in cell proliferation, primary hepatocytes from WT and β_2_ARKO mouse livers were isolated and cultured. In β_2_ARKO hepatocytes, HGF- and βAR agonist clenbuterol-induced cell proliferation was blocked compared to WT hepatocytes according to the CCK-8 assay (Fig. 5A, B). Moreover, as shown in Fig. 5C, adenovirus-mediated β_2_AR overexpression was found to promote hepatocyte proliferation in both WT and β_2_ARKO hepatocytes. In line with these findings, the number of EdU-positive nuclei was increased by virus infection in the presence or absence of HGF (Fig. 5D).

Next, we investigated the underlying mechanisms of the decreased cell proliferation phenotypes in β_2_ARKO hepatocytes. Expression levels of checkpoint components of the cell cycle machinery were measured at different time points in response to HGF treatment. Interestingly, we found that in WT hepatocytes, the level of cyclin E1 was increased in a time-dependent manner induced by HGF, while it was unchanged in β_2_ARKO hepatocytes. Furthermore, loss of β_2_AR led to a delay in CDK2 and PCNA induction in response to HGF (Fig. 5E). We also examined MAPK/ERK and AKT signaling in WT and β_2_ARKO hepatocytes. As shown in Fig. 5F, the lack of β_2_AR caused a decrease in the phosphorylation of ERK and AKT at several early time points (Fig. 5F). Together, these findings indicated that β_2_AR played a crucial role in hepatocyte proliferation.

### β_2_AR signaling and c-met signaling crosstalk during liver regeneration

**Fig. 6:**
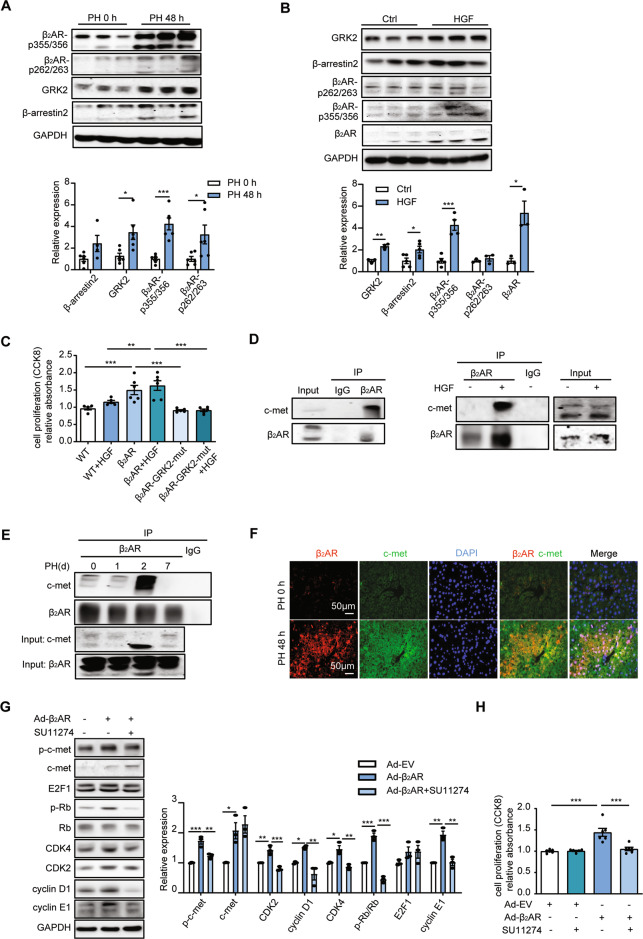
β_2_AR signaling was associated with c-met signaling during liver regeneration. **A** Western blot analysis of the protein levels of the β_2_AR signaling cascade in mouse livers 48 h post PH (*n* = 4–6). **B** Western blot analysis of the protein levels of the β_2_AR signaling cascade in hepatocytes treated with HGF for 24 h. **C** Primary hepatocytes were treated with β_2_AR or β_2_AR-GRK2-mut virus in the presence or absence of HGF, and then a CCK-8 assay was performed. **D** Coimmunoprecipitation assays demonstrated that β_2_AR could bind to c-met in primary hepatocytes, and the binding of the two receptors was significantly increased in response to HGF treatment. **E** Coimmunoprecipitation assay showed the combination of β_2_AR with c-met at the indicated time post PH. **F** Immunofluorescence of β_2_AR, c-met and DAPI in mouse livers post PH. **G** Western blot analysis of the protein levels of cell cycle markers in hepatocytes treated with β_2_AR virus in the presence or absence of the c-met inhibitor SU11274. **H** Hepatocytes treated with β_2_AR virus in the presence or absence of the c-met inhibitor SU11274, and then a CCK-8 assay was performed. Data are shown as means ± SEM. *P < 0.05, **P < 0.01, ***P < 0.001 with a two-tailed Student’s t test in (**A**, **B**). *P < 0.05, **P < 0.01, ***P < 0.001 by one-way ANOVA and post hoc Tukey’s test in (**C**, **G**, **H**).

Our previous studies suggested the existence of a novel insulin receptor-induced and G-protein-coupled receptor kinase 2 (GRK2)-mediated transactivation of a β_2_AR-β-arrestin2-ERK signaling cascade that was the primary mediator of insulin-induced ERK activation in cardiomyocytes [22]. Inspired by these findings, we determined the GRK2-β_2_AR-β-arrestin2 signaling cascade 48 h post PH. Notably, GRK2-mediated activation of a β_2_AR-β-arrestin2 signaling cascade was observed in livers post PH (Fig. 6A). We further examined the signaling cascade in primary hepatocytes after HGF treatment. Indeed, the levels of GRK2-mediated phosphorylation of β_2_AR and β-arrestin2 expression were significantly increased by HGF (Fig. 6B). Moreover, in primary hepatocytes, introduction of β_2_AR but not mutant β_2_AR lacking GRK2 phosphorylation sites promoted hepatocyte proliferation in the presence or absence of HGF (Fig. 6C). Since HGF/c-met signaling is an essential growth factor signaling pathway during liver regeneration [6], these data supported the existence of a link between β_2_AR signaling and HGF/c-met signaling. Thus, we determined the HGF levels in the serum and liver between WT and β_2_ARKO mice post PH. Interestingly, no difference was found between these two groups (Fig. S1D–F), which prompted us to consider whether β_2_AR could form a complex with the HGF receptor (c-met) to regulate downstream signaling pathways. As shown in Fig. 6D, β_2_AR could bind to c-met in primary hepatocytes, and the binding of the two receptors was significantly increased after HGF treatment. Consistently, in regenerating livers, a large number of β_2_AR and c-met complexes were noticed 2 days post-PH, while during the termination of liver regeneration, they disassociated from each other (Fig. 6E). Furthermore, immunofluorescence staining showed colocalization of β_2_AR and c-met in the nucleus in mouse livers after surgery (Fig. 6F).

To further investigate the relationship of β_2_AR and the HGF receptor c-met, we determined the phosphorylation of c-met in β_2_AR-overexpressed hepatocytes. Interestingly, the protein levels of p-c-met and c-met were both upregulated after β_2_AR introduction. Meanwhile, the expression levels of CDK2, CDK4, p-Rb/Rb and cyclin E1 were significantly increased upon β_2_AR virus treatment (Fig. 6G). Furthermore, the upregulation of these cell cycle markers and cell proliferation was abolished when c-met was inhibited with SU11274 (Fig. 6G–H). These data suggested a crosstalk between β_2_AR and c-met signaling during liver regeneration.

### β_2_AR-mediated hepatocyte proliferation was ERK-dependent

**Fig. 7:**
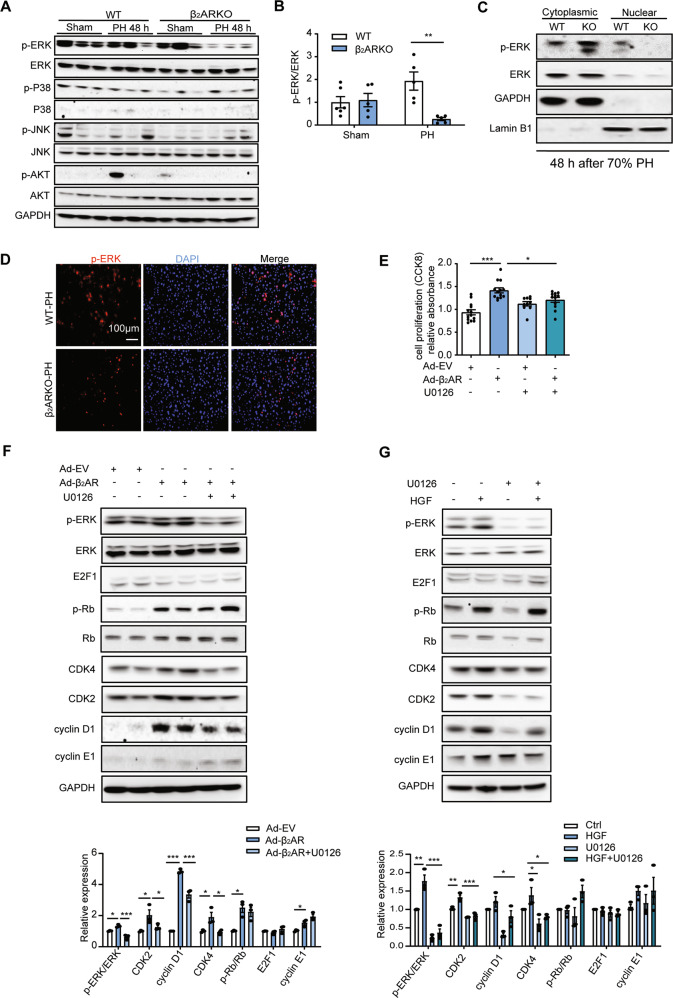
β_2_AR-mediated cell proliferation was partially dependent on ERK signaling. **A** The protein levels of p-ERK/ERK, p-P38/P38, p-JNK/JNK and p-AKT/AKT in β_2_ARKO and WT mouse livers 48 h post PH. **B** Quantitative analysis of immunoblotting (*n* = 5–6). **C** Immunoblotting for cytoplasmic and nuclear p-ERK and ERK in β_2_ARKO and WT mouse livers 48 h post PH. **D** Immunofluorescence of p-ERK demonstrated that p-ERK failed to enter the nucleus in β_2_ARKO mouse livers post PH. Data are shown as means ± SEM. *P < 0.05, **P < 0.01, ***P < 0.001 with a two-tailed Student’s t test in (**B**). **E** Primary hepatocytes were treated with β_2_AR virus in the presence or absence of the ERK inhibitor U0126, and then a CCK-8 assay was performed. **F** Western blot analysis of the protein levels of cell cycle markers in hepatocytes treated with β_2_AR virus in the presence or absence of the ERK inhibitor U0126. **G** Western blot analysis of the protein levels of cell cycle markers in hepatocytes pretreated with U0126 in the presence or absence of HGF. Data are shown as means ± SEM. *P < 0.05, **P < 0.01, ***P < 0.001 by one-way ANOVA and post hoc Tukey’s test in (**E**–**G**).

Cell cycle arrest in the proliferating hepatocytes of β_2_ARKO mice prompted us to consider whether a growth factor signaling pathway was disrupted, since β_2_ARKO mice showed a similar phenotype to that of mice lacking c-met [6]. Thus, we analyzed the most important downstream pathways of growth factor receptors, including the MAPK/ERK cascade, SAPK/JNK cascade, P38-MAPK cascade and Akt signaling. Notably, the levels of p-ERK/ERK were reduced in β_2_ARKO mouse livers postsurgery, while the levels of p-P38/P38, p-JNK/JNK and p-AKT/AKT were not significantly changed (Figs. 7A, B and S1C). In addition, Western blotting and immunofluorescence showed that the levels of p-ERK in the nucleus were decreased in β_2_ARKO mouse livers 48 h post PH (Fig. 7C, D). Together, these results indicated that loss of β_2_AR impaired the MAPK/ERK pathway during liver regeneration.

To further explore the role of ERK signaling in β_2_AR-induced cell proliferation, we next disrupted this signaling with the specific inhibitor U0126 in β_2_AR-overexpressing hepatocytes. A Cell Counting Kit-8 (CCK-8) assay indicated that β_2_AR-mediated cell proliferation was prevented by U0126 treatment (Fig. 7E). Similar to the results obtained in vivo, the expression levels of CDK2, CDK4, p-Rb/Rb and cyclin E1 were significantly increased upon β_2_AR virus treatment, while only CDK2 and CDK4 were reduced by ERK inhibition (Fig. 7F). Moreover, HGF-induced CDK2 and CDK4 expression was reduced by ERK inhibition (Fig. 7G). These data suggested that β_2_AR-induced cell proliferation was partly dependent on ERK activation.

## Discussion

Previous findings have shown the great significance of α1-adrenergic receptors in the regulation of liver regeneration [23]. The effect of nonselective β-blocker propranolol and highly selective β_1_-receptor antagonist nebivolol on liver regeneration has been studied but is controversial [24–26], which may be related to the dosage form, time of administration, dosage, method of administration and drug selectivity and so on. However, these studies did not really clarify the role of β_2_AR in regenerating livers and proliferating hepatocytes. In our study, β_2_AR gene knockout mice and AAV8-β_2_AR were used to further investigate the loss- and gain-of function effects of β_2_AR on liver regeneration. In future studies, liver-specific gene knockout mice will be used to reinforce our conclusions. In this study, we demonstrate that β_2_AR is a critical factor contributing to liver regeneration. β_2_AR deficiency leads to a low survival rate after PH. Moreover, both loss- and gain-of-function studies revealed that β_2_AR is necessary for regular cell cycle progression and proliferation of hepatocytes. Mechanistically, GRK2-mediated activation of β_2_AR recruits the HGF receptor (c-met) complex to initiate the phosphorylation of ERK and subsequent cell proliferation. Finally, mutation of the GRK2 phosphorylation site on β_2_AR or ERK inhibition robustly disrupted hepatocyte proliferation mediated by Ad-β_2_AR or HGF treatment. Therefore, β_2_AR promotes liver regeneration partially through crosstalk with c-met and subsequent activation of ERK signaling. Besides, nuclear transfer of β_2_AR during liver regeneration was found in this study, and β_2_AR can bind and phosphorylate hepatocyte growth factor receptor c-met, which has not been reported. Furthermore, how β_2_AR transposes and regulates downstream signals needs further study. The research results obtained in this project will expand the understanding of the regulation mechanism of liver regeneration and provide new targets and new ideas for the diagnosis and treatment of related liver diseases.

Early lipid accumulation is a hallmark of liver regeneration, and fatty acids serve as an energy source during the initial steps of regeneration [27]. Moreover, another study showed that transient attenuation of hepatic lipid accumulation in response to propranolol treatment impaired liver regeneration after PH [25]. Consistently, in our present study, β_2_ARKO mice displayed a significant reduction in lipid formation post-PH compared to WT mice. In addition, glucose availability is another key factor during liver regeneration [28] and it has been suggested that inactivation of β_2_AR disrupted glucose homeostasis in mice [29]. Together, these findings suggest that aberrant hepatic energy status may be associated with high mortality and disrupted liver regeneration in β_2_ARKO mice.

Liver regeneration is known to be a process involving highly organized and ordered tissue growth upon surgical resection and following viral or drug-induced liver injury. In contrast to all other organs, liver-to-body-weight ratio needs to be maintained always at 100% of what is required for body homeostasis [30]. A large number of genes are involved in liver regeneration, and the overall process includes three phases. The initial step is characterized by priming of quiescent hepatocytes by factors such as TNF-α and IL-6. The proliferative stage is the step during which hepatocytes enter into the cell cycle’s G1 phase with the stimulation of complete mitogens including HGF, TGF-α and EGF. Non-mitogenic cytokines, insulin, TNF-α, bile acids and IL-6 have also been implicated in regulating the regeneration process. To avoid excessive liver regeneration, there are many signals associated with regeneration termination, such as C/EBP transcription factors and Glypican-3 [4]. Therefore, in our study, we suggested that β_2_AR accelerated liver regeneration during the proliferation step, but it did not increase the final level of liver regeneration.

In proliferating cells, E2F1 dissociates from Rb before translocating to the nucleus when Rb is phosphorylated and then initiates the transcription of cyclin E1 and other cell cycle markers [31]. Cyclin E1 interacts with CDK2 to form a complex that promotes the G1/S transition [21]. Our data, both in vivo and in vitro, demonstrated that β_2_AR signaling might regulate hepatocyte proliferation by modulating the G1/S transition. Furthermore, some of the protein changes that were not as expected could be attributed to the choice of processing timing.

Movement of plasma membrane receptors to the nucleus has also been described for some other GPCRs, for receptor tyrosine kinases (RTKs), and in various cell types, which have been reported to play essential roles in cell proliferation [6, 32–35]. A similar phenomenon of β_2_AR has been observed in liver regeneration, suggesting that there is a link between β_2_AR translocation and hepatocyte proliferation. Our previous study showed that the insulin receptor induces arrestin-biased transactivation of β_2_AR in a GRK2 phosphorylation-dependent manner [22]. Previous research has also uncovered a novel function for c-met in regulating hepatic glucose metabolism by directly interacting with and regulating the insulin receptor [36]. Inspired by these findings, our data demonstrate crosstalk between β_2_AR signaling and c-met signaling. However, the possibility of forming a complex of these three receptors in the regulation of liver regeneration could not be ruled out. Since β_2_AR-mediated epidermal growth factor receptor (EGFR) transactivation in DNA synthesis has been well documented in recent studies [37–39], the association of β_2_AR with other RTKs, including EGFR, post PH should be further explored.

The MAPK/ERK pathway, which regulates cell proliferation, displayed significant disruption in β_2_AR-deficient livers after PH and hepatocytes in response to HGF treatment. β_2_AR-mediated cell proliferation is prevented by U0126 treatment. Furthermore, β_2_AR-mediated upregulation of most of the cell cycle markers can be reduced by ERK inhibition. Nevertheless, the expression manner of p-Rb/Rb, E2F1 and cyclin E1 in response to β_2_AR and U0126 treatment is complicated, which demonstrates that β_2_AR-mediated cell proliferation is partially dependent on ERK signaling, and the manner by which these signaling pathways synergistically promote subsequent cell cycle progression is much more complicated than we have so far understood.

**Fig. 8:**
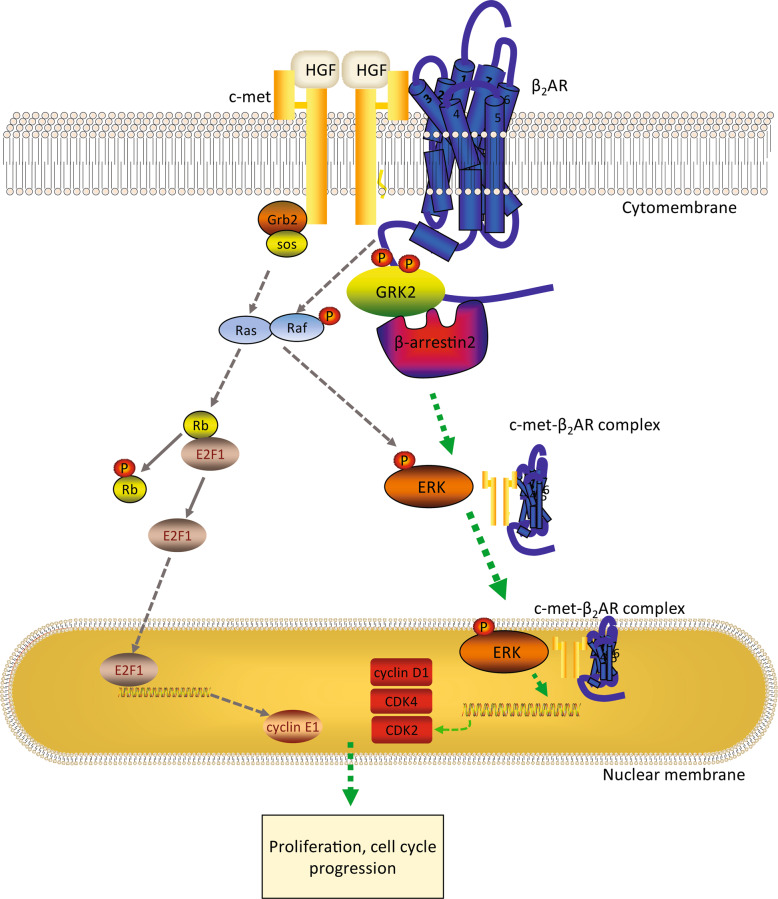
β2-adrenergic receptor promotes liver regeneration partially through crosstalk with c-met. Model of β_2_AR as a critical factor that regulates hepatocyte proliferation during liver regeneration.

In conclusion, our study provides new insights into the dynamic nature of β_2_AR in the regulation of liver regeneration, as presented in Fig. 8. Our findings highlight the importance of β_2_AR in the promotion of cell cycle progression in proliferating cells and support the β_2_AR-c-met complex as a novel therapeutic target for abnormal proliferative diseases, such as benign and malignant tumors.

## Materials and methods

### Animals and treatments

Animal experiments were performed following the National Institutes of Health Guide for the Care and Use of Laboratory Animals. All animal studies were approved by the Animal Experimentation Ethics Committee of Huazhong University of Science and Technology. Eight-week-old male C57BL/6J mice (HFK Bioscience, Beijing, China) and β_2_ adrenergic receptor (β_2_AR) global knockout mice (a gift from Dr. Yang K. Xiang, UC Davis, USA) were challenged by 70% partial hepatectomy (PH). All mice were housed under a 12:12 h light/dark cycle at controlled temperature.

For PH, mice (*n* = 21 per group) received food and water *ad libitum*. PH was performed under 2,2,2-tribromoethanol anesthesia. Three liver lobes were removed [40]. As a control, sham operations were performed, but the livers were not removed (*n* = 6 per group). Animals were randomly assigned to sham or PH group. Mice were killed, and liver samples were harvested at different time points specified in each experiment. The hepatic index (liver weight/body weight) was monitored for each mouse over 7 days.

### Human tissues

All human tissue samples were obtained by surgery from Tongji Hospital of Tongji medical college and informed consents were obtained from the patients. All study methodologies were strictly in accordance with Helsinki declaration for the Use of Human Subjects and were approved by the Ethics Committee at Tongji Medical College, Huazhong University of Science and Technology.

### Cell culture and treatments

Primary hepatocytes were isolated by a collagenase IV (0.5 mg/ml, Worthington, NJ) perfusion method as previously described [41]. Hepatocytes were treated with hepatocyte growth factor (HGF, 40 ng/mL) for the indicated hours to mimic an in vivo model of 70% PH. The c-met inhibitor SU11274 (Selleck, Shanghai, China), ERK inhibitor U0126 (Selleck, Shanghai, China) and βAR agonist clenbuterol (Selleck, Shanghai, China) were used at 1, 10, and 1 μM, respectively. The adenovirus encoding β_2_AR (Ad-β_2_AR) (a gift from Dr. Yang K. Xiang, UC Davis, USA) was used to overexpress β_2_AR in vitro.

### Cell proliferation assay

Cell growth was determined using the CCK-8 assay (Cell Counting Kit-8, Dojindo, Japan) following the manufacturer’s instructions. Briefly, primary hepatocytes seeded in 96-well plates were treated with HGF for 24 h. CCK-8 (10%) was added to the culture medium for 2 h, and then the absorbance value was measured at 450 nm.

### Histological analysis

Liver tissues were fixed in formalin. Sections were stained with H&E and Ki67. Hepatic lipid content was analyzed on frozen sections of livers by Oil Red O staining (O0625, Sigma, St. Louis, MO) and counterstained with Mayer’s hematoxylin.

### AAV8-mediated overexpression of β_2_AR in mouse liver

To overexpress β_2_AR in mouse livers, recombinant β_2_AR plasmids were driven by a liver-specific promoter (thyroxine-binding globulin, TBG) and packaged into adeno-associated virus 8 (AAV8) particles (Genechem, Shanghai, China). A total of 1 × 10^11^ plaque-forming units (pfu) of AAV8-β_2_AR or AAV8-GFP in 200 µl saline were injected into mice via the tail vein, and 4 weeks later, 70% PH was performed.

### Western blot analysis

Tissue extracts or mouse primary hepatocytes were lysed using RIPA buffer, and total protein was extracted and separated by SDS–PAGE and then transferred onto a PVDF membrane. Membranes were probed with antibodies. Antibodies used in this study are listed in Table S1. Chemiluminescent detection was performed with horseradish peroxidase-coupled secondary antibody (Cell Signaling, Danvers, MA) and Super Signal West Femto reagent (Servicebio, Wuhan, China). Band densities were quantified using ImageJ software.

### RNA extraction and quantitative real-time PCR analysis

Total RNA was isolated with TRI Reagent (D9108A, Takara Bio). cDNA was synthesized using an RNA PCR Kit (RR036A, Takara Bio) and analyzed by real-time PCR using SYBR Green Premix Ex Taq (Takara, Japan). The primers for the PCR and siRNA targeting sequences used in this study are listed in Table S2.

### Statistical analysis

Data were tested for normality using Shapiro–Wilk and Kolmogorov–Smirnov normality tests. All normally distributed data were analyzed using Student’s t test, one-way ANOVA, followed by Tukey’s post hoc analysis. For data not normally distributed, statistical analyses were performed using the non-parametric Kruskal–Wallis test. All experiments were performed at least in triplicate, and representative data are shown. All analysis was performed using Prism (GraphPad Software, San Diego, CA). *P* < 0.05 was defined as statistically significant.

## Supplementary information

Figure S1

Supplementary figure legends and table

checklist

Original Data File

## Data Availability

The datasets generated and analyzed during the current study are available from the corresponding author on reasonable request.
