# Supplementary figures and images for: β2-adrenergic receptor promotes liver regeneration partially through crosstalk with c-met
Source: Cell Death Dis. 2022 Jun 27;13(6):571. doi: 10.1038/s41419-022-04998-0 (PMC9237079; doi:10.1038/s41419-022-04998-0)

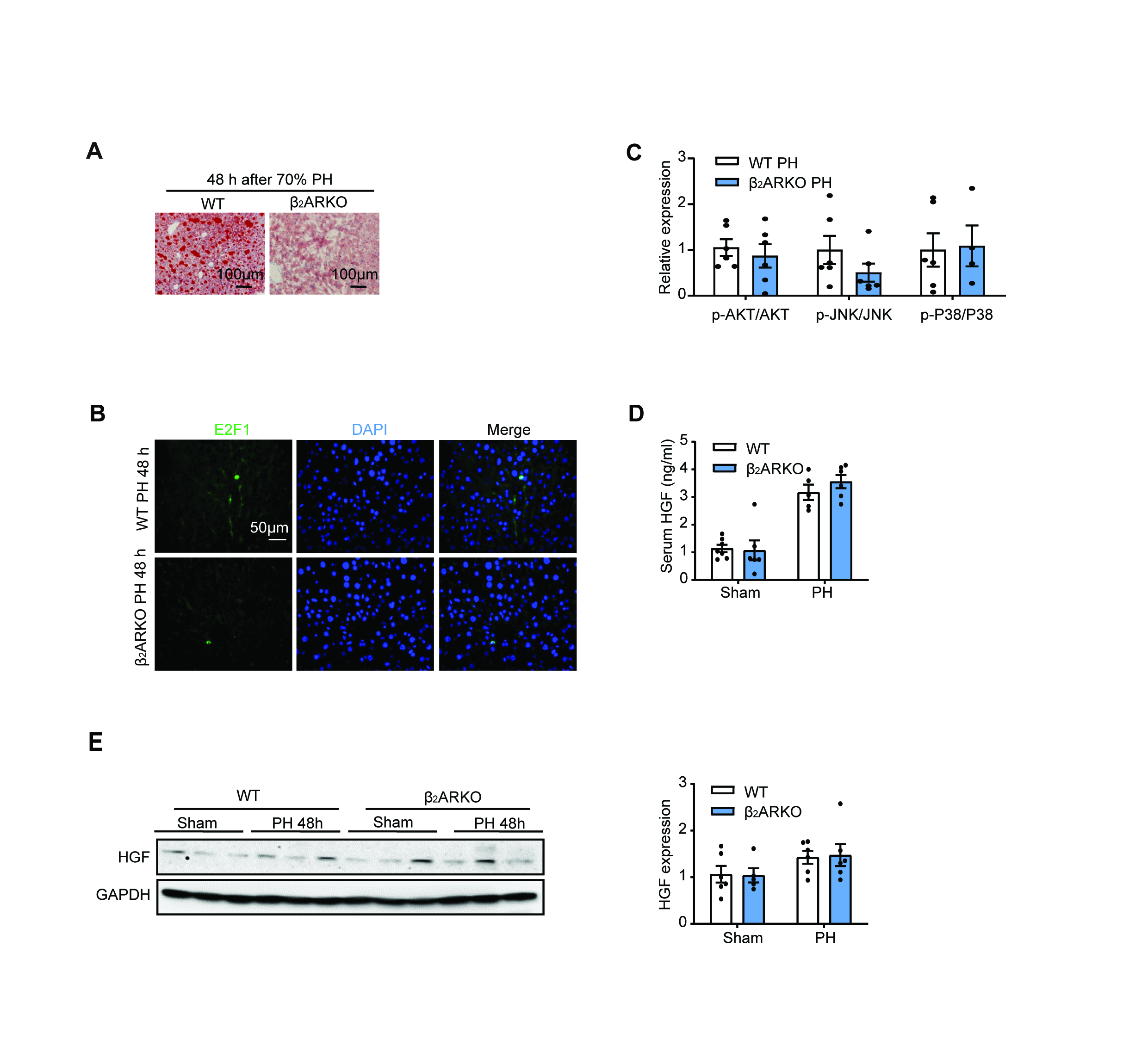

Supplement: Supplementary file 1 — Figure S1 [file 41419_2022_4998_MOESM1_ESM.tif]
