# Supplementary material for: β2-adrenergic receptor promotes liver regeneration partially through crosstalk with c-met
Source: Cell Death Dis. 2022 Jun 27;13(6):571. doi: 10.1038/s41419-022-04998-0 (PMC9237079; doi:10.1038/s41419-022-04998-0)
Supplement: Supplementary file 2 — Supplementary figure legends and table [file 41419_2022_4998_MOESM2_ESM.doc]

**Online Supplementary Data**

Table S1. Antibodies used in the study

| Antibodies | Product code | Company |
| --- | --- | --- |
| Anti-β_1_AR | sc-568 | Santa Cruz |
| Anti-β_2_AR | A5461 | Bimake |
| Anti-β_2_AR | sc-569 | Santa Cruz |
| Anti-β_2_AR | sc-570 | Santa Cruz |
| Anti-β-arrestin2 | sc-13140 | Santa Cruz |
| Anti-β_3_AR | sc-515763 | Santa Cruz |
| Anti-GRK2 | sc-562 | Santa Cruz |
| Anti-p-P38 MAPK | 9216 | Cell signaling |
| Anti-P38 MAPK | 9212 | Cell signaling |
| Anti-p-SAPK/JNK | 9255 | Cell signaling |
| Anti-SAPK/JNK | 9252 | Cell signaling |
| Anti-p-ERK | 4370 | Cell signaling |
| Anti-ERK | 4695 | Cell signaling |
| Anti-p-AKT | 4060 | Cell signaling |
| Anti-AKT | 9272 | Cell signaling |
| Anti-cyclin B1 | 55004-1-AP | Proteintech |
| Anti-cyclin D1 | A5035 | Bimake |
| Anti-cyclin E1 | 11554-1-AP | Proteintech |
| Anti-CDK2 | 10122-1-AP | Proteintech |
| Anti-CDK6 | 14052-1-AP | Proteintech |
| Anti-E2F1 | A5693 | Bimake |
| Anti-c-met | 3127 | Cell signaling |
| Anti-CDK4 | 11026-1-AP | Proteintech |
| Anti-PCNA | 10205-2-AP | Proteintech |

Table S2. Oligonucleotides used in the study

| Target gene | Forward sense | Reverse sense |
| --- | --- | --- |
| β_1_AR | CTACAACGACCCCAAGTGCT | GGCACGTAGAAGGAGACGAC |
| β_2_AR | GGGAACGACAGCGACTTCTT | GCCAGGACGATAACCGACAT |
| β_3_AR | AGAAACGGCTCTCTGGCTTTG | TGGTTATGGTCTGTAGTCTCGG |

Supplementary figure legends

Figure S1. Characterization of liver in WT and β_2_ARKO mice post-PH.

(A) Oil red O staining of WT and β_2_ARKO mice liver 48 h after 70% PH. (B) Immunostaining of E2F1 in WT and β_2_ARKO mice liver 48 h after 70% PH. (C) The quantitative analysis of immunoblotting (n=4-6). (D) Serum HGF levels in indicated groups (n=5-7). (E) The protein levels of HGF in β_2_ARKO and WT mice liver 48 h post-PH (n=5-6).
