# Supplementary material for: β2-adrenergic receptor promotes liver regeneration partially through crosstalk with c-met
Source: Cell Death Dis. 2022 Jun 27;13(6):571. doi: 10.1038/s41419-022-04998-0 (PMC9237079; doi:10.1038/s41419-022-04998-0)
Supplement: Supplementary file 4 — Original Data File [file 41419_2022_4998_MOESM4_ESM.pptx]

## Slide 1
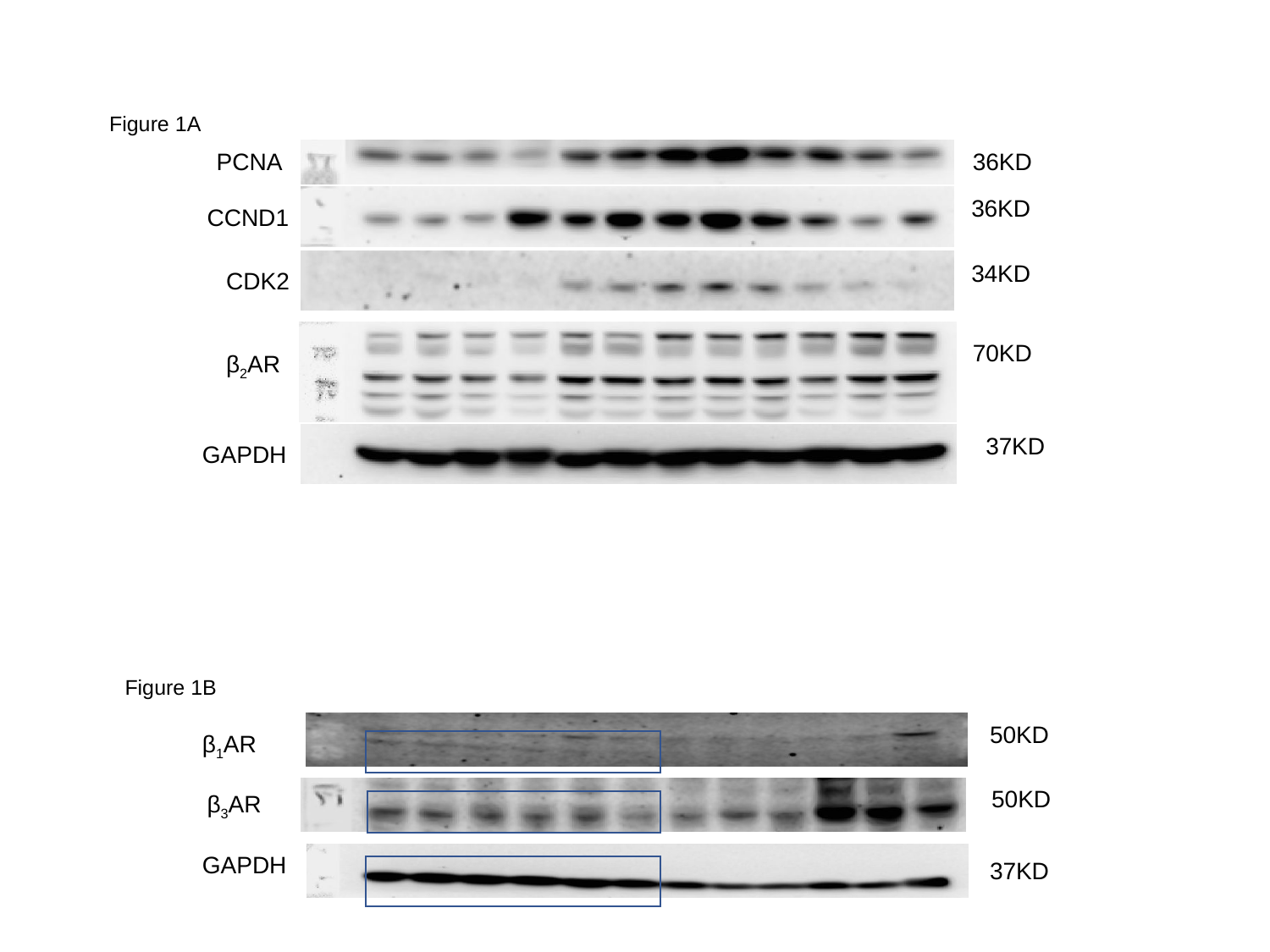

Figure 1A
PCNA
36KD
36KD
CCND1
34KD
CDK2
70KD
β2AR
37KD
GAPDH
Figure 1B
50KD
β1AR
50KD
β3AR
GAPDH
37KD

## Slide 2
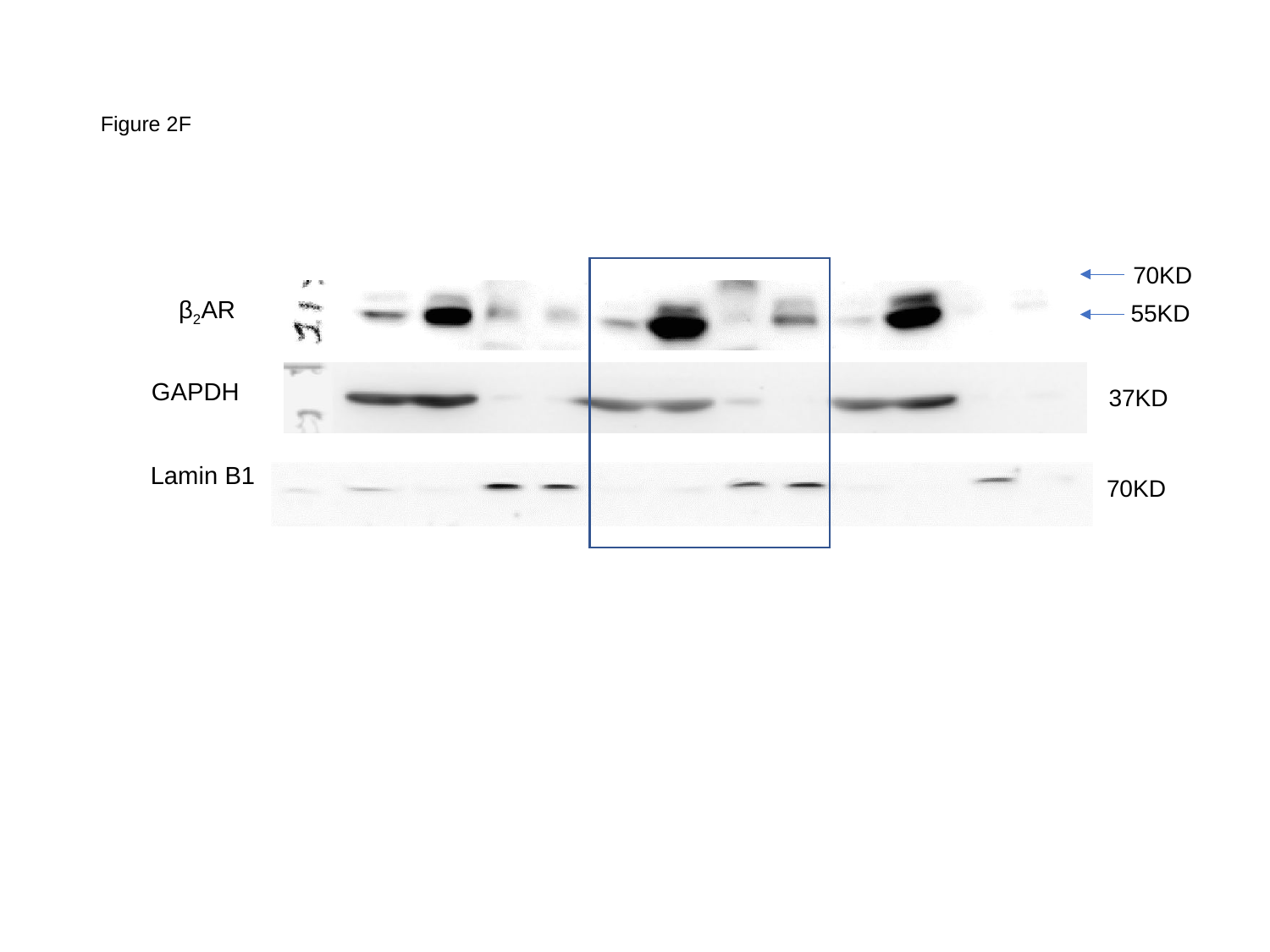

Figure 2F
70KD
β2AR
55KD
GAPDH
37KD
Lamin B1
70KD

## Slide 3
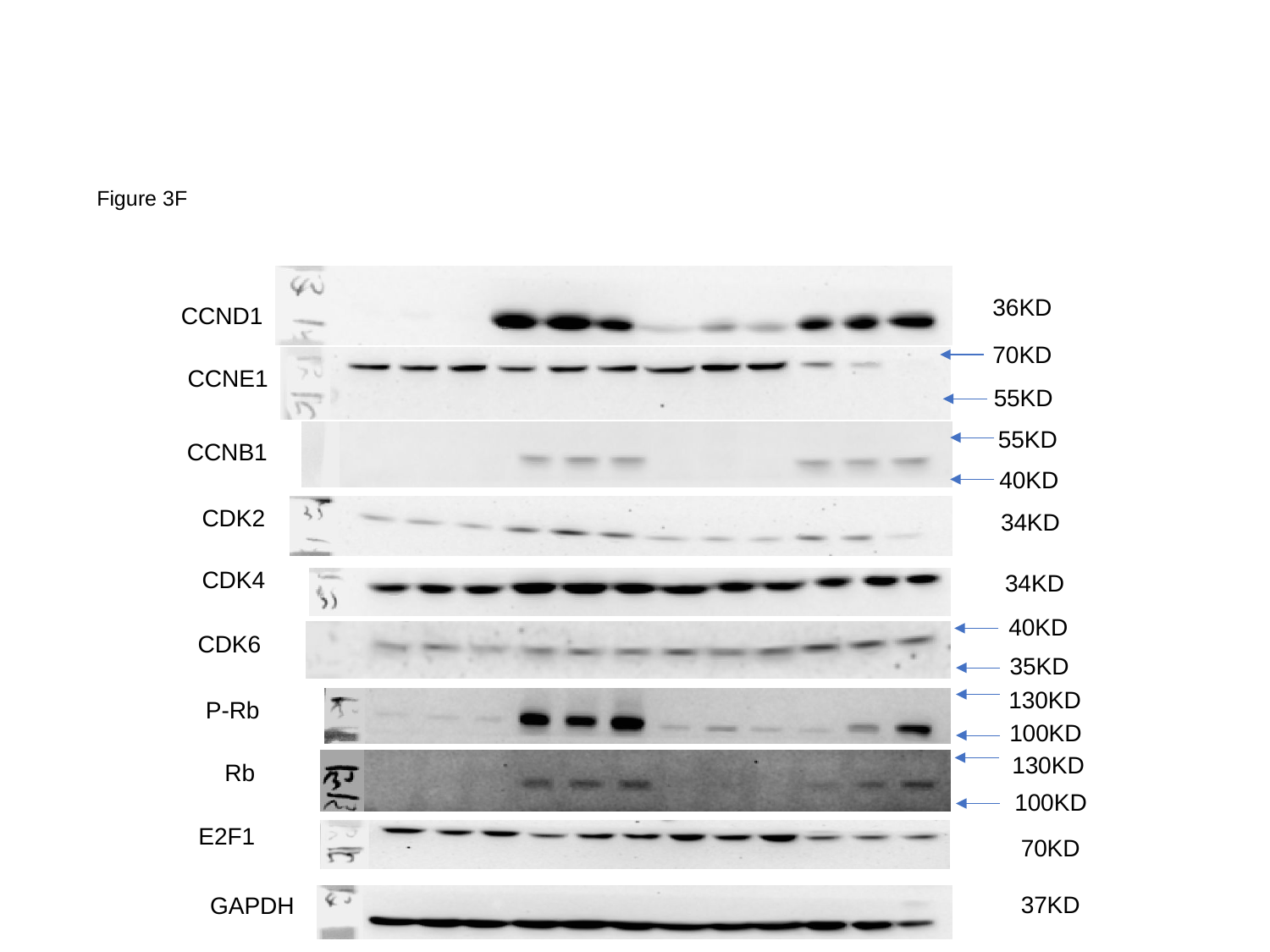

Figure 3F
36KD
CCND1
70KD
CCNE1
55KD
55KD
CCNB1
40KD
CDK2
34KD
CDK4
34KD
40KD
CDK6
35KD
130KD
P-Rb
100KD
130KD
Rb
100KD
E2F1
70KD
37KD
GAPDH

## Slide 4
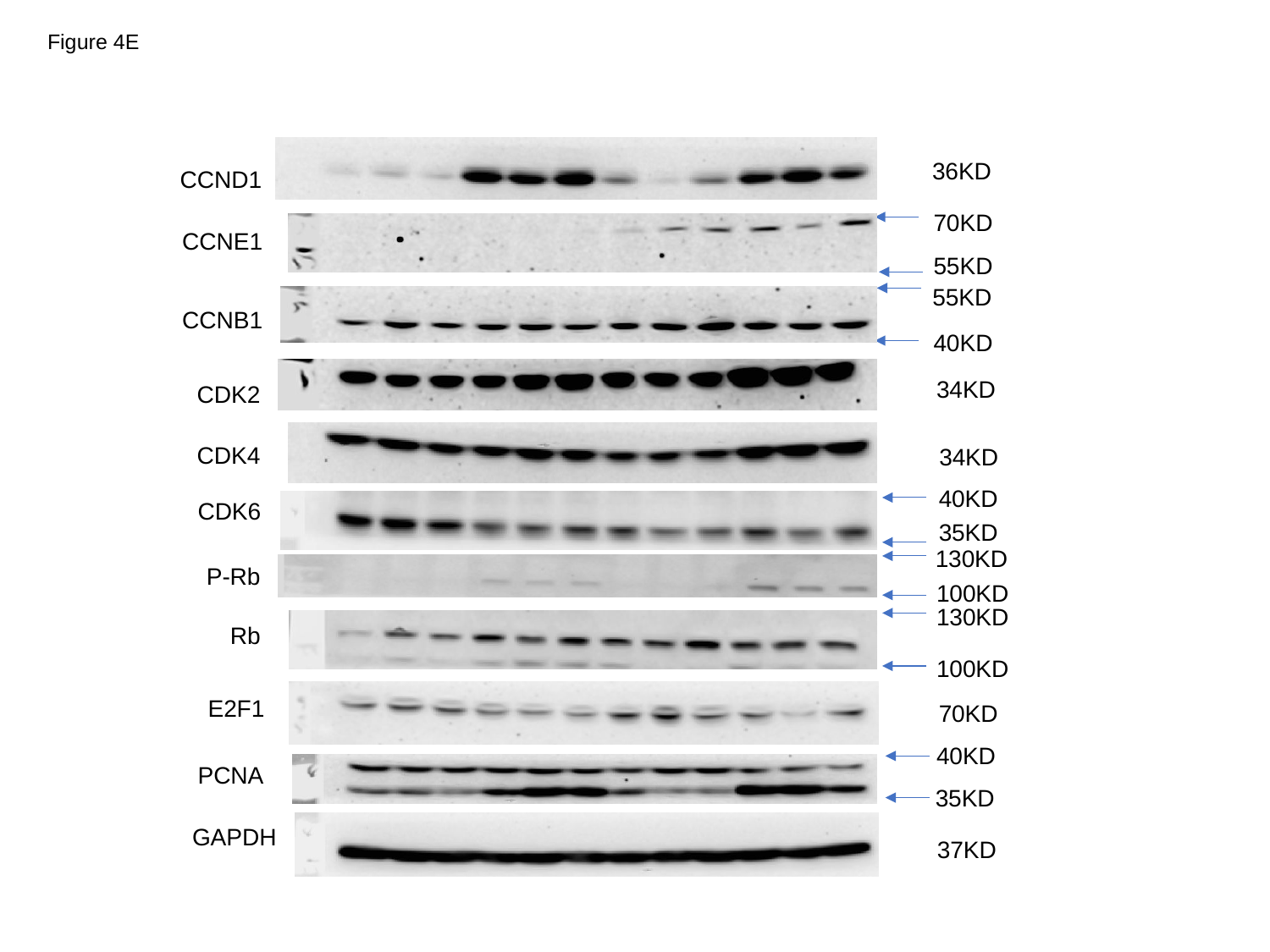

# Figure 4E
36KD
CCND1
70KD
CCNE1
55KD
55KD
CCNB1
40KD
34KD
CDK2
CDK4
34KD
40KD
CDK6
35KD
130KD
P-Rb
100KD
130KD
Rb
100KD
E2F1
70KD
40KD
PCNA
35KD
GAPDH
37KD

## Slide 5
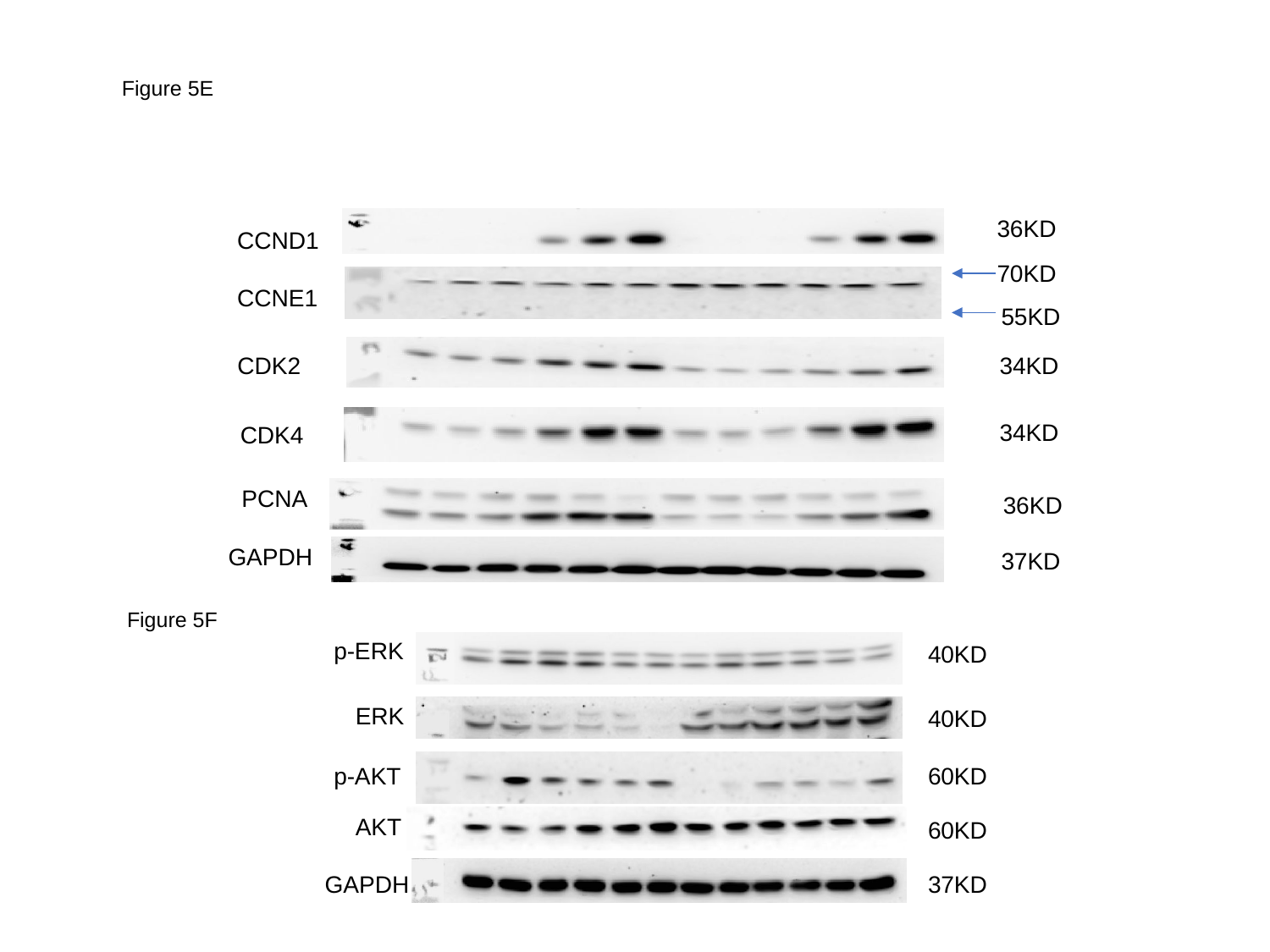

# Figure 5E
36KD
CCND1
70KD
CCNE1
55KD
CDK2
34KD
34KD
CDK4
PCNA
36KD
GAPDH
37KD
Figure 5F
p-ERK
40KD
ERK
40KD
p-AKT
60KD
AKT
60KD
GAPDH
37KD

## Slide 6
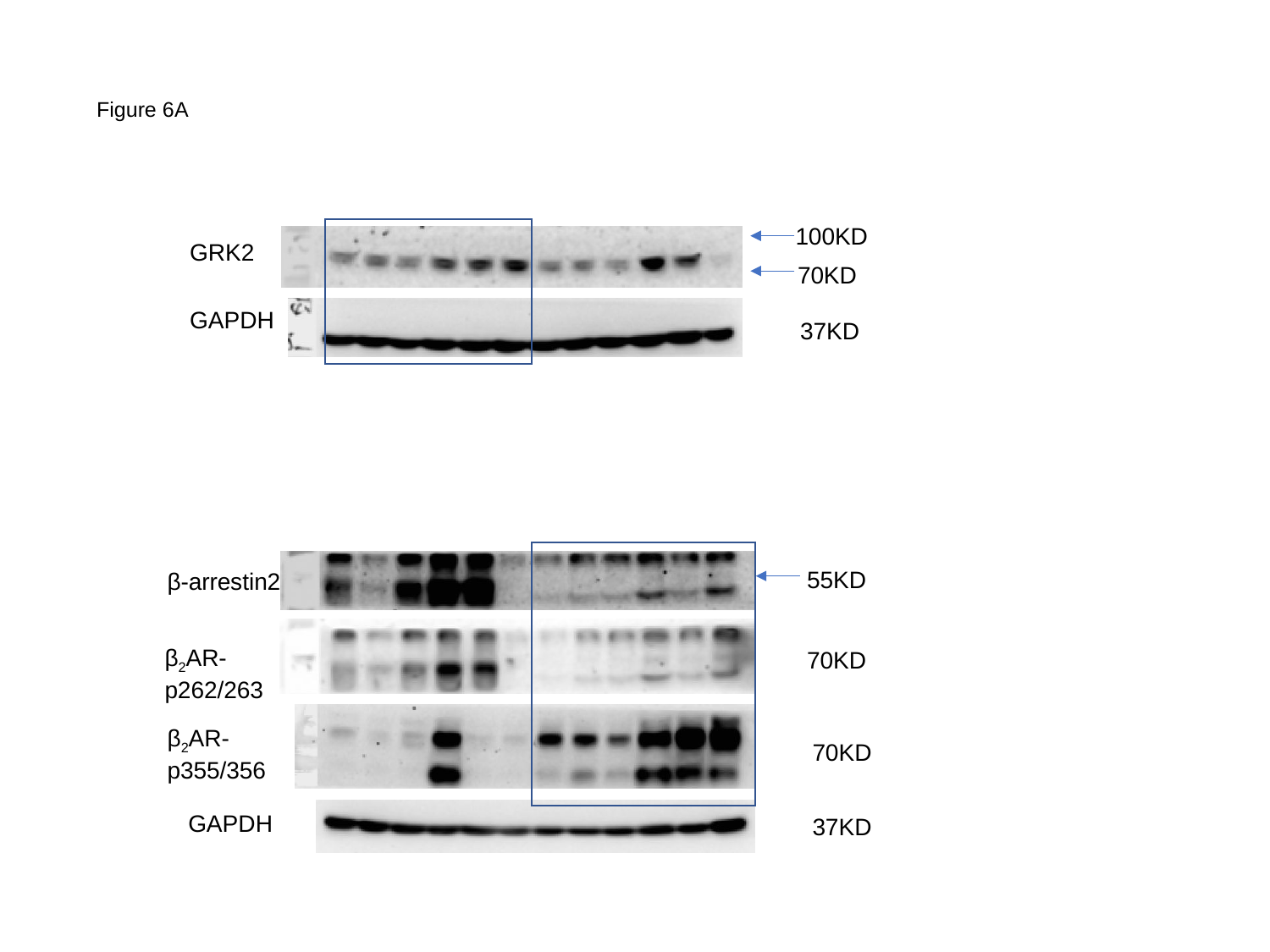

# Figure 6A
100KD
GRK2
70KD
GAPDH
37KD
55KD
β-arrestin2
β2AR-p262/263
70KD
β2AR-p355/356
70KD
GAPDH
37KD

## Slide 7
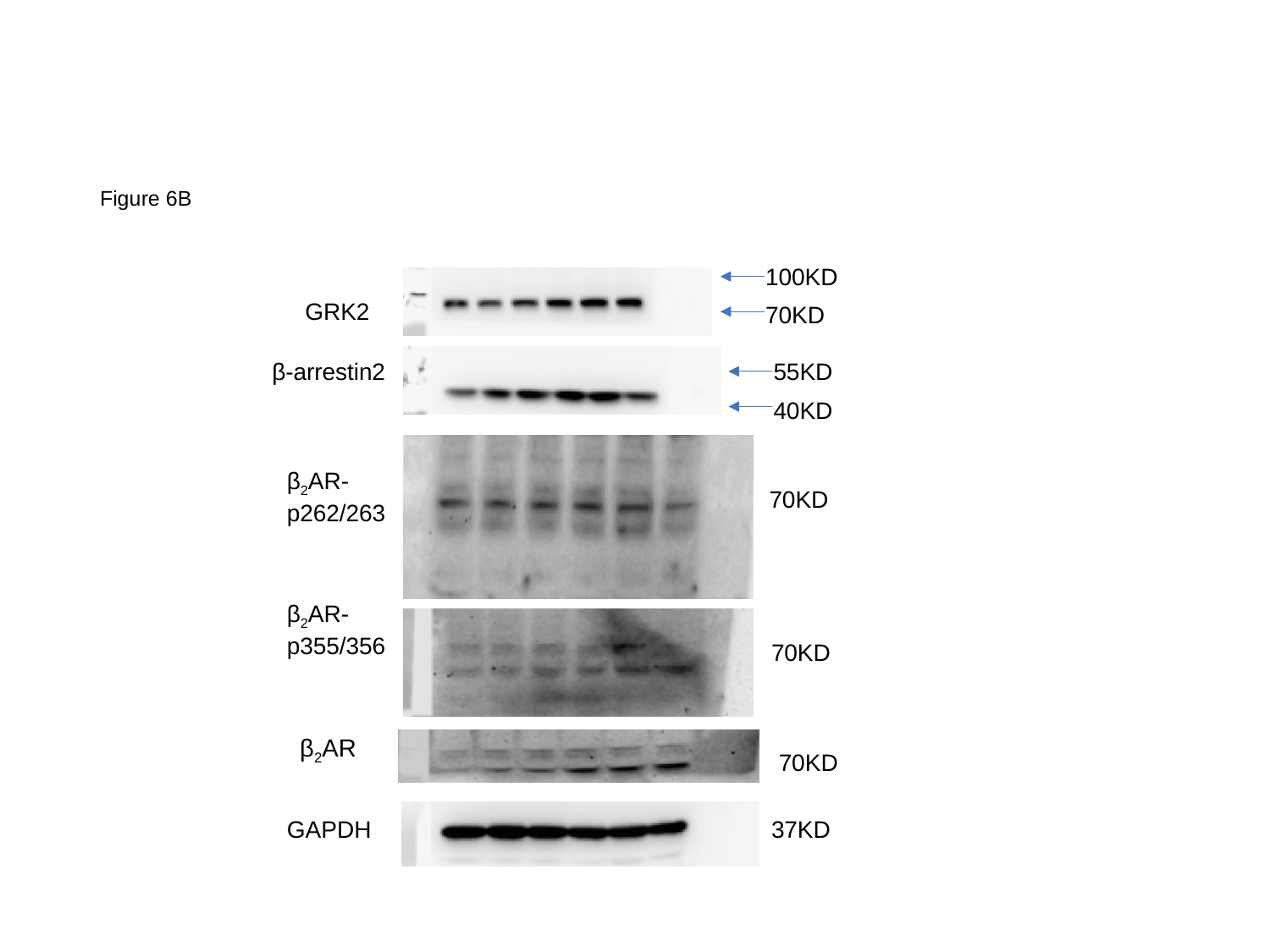

# Figure 6B
100KD
GRK2
70KD
β-arrestin2
55KD
40KD
β2AR-p262/263
70KD
β2AR-p355/356
70KD
β2AR
70KD
GAPDH
37KD

## Slide 8
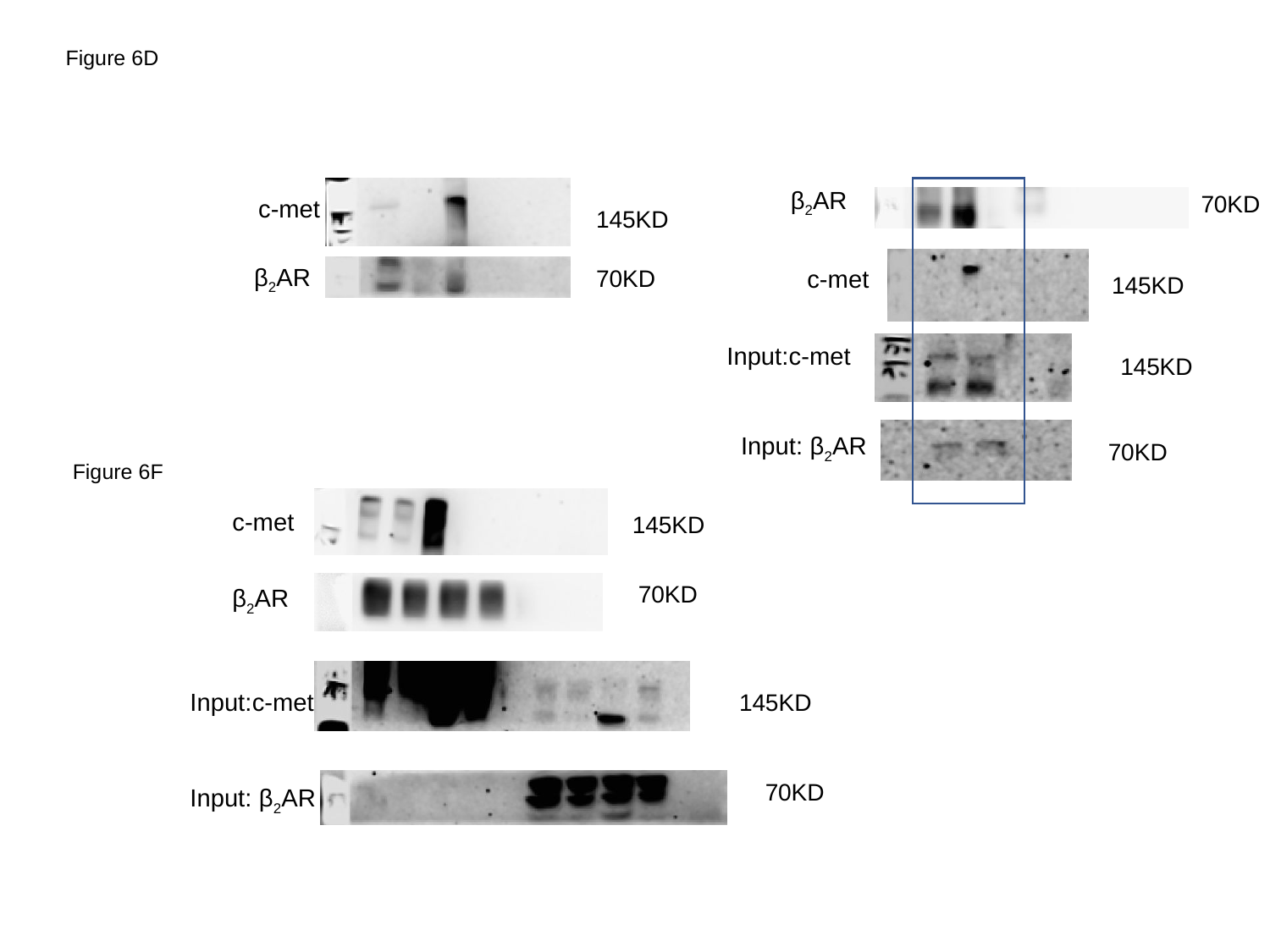

Figure 6D
β2AR
70KD
c-met
145KD
β2AR
70KD
c-met
145KD
Input:c-met
145KD
Input: β2AR
Figure 6F
70KD
c-met
145KD
70KD
β2AR
Input:c-met
145KD
70KD
Input: β2AR

## Slide 9
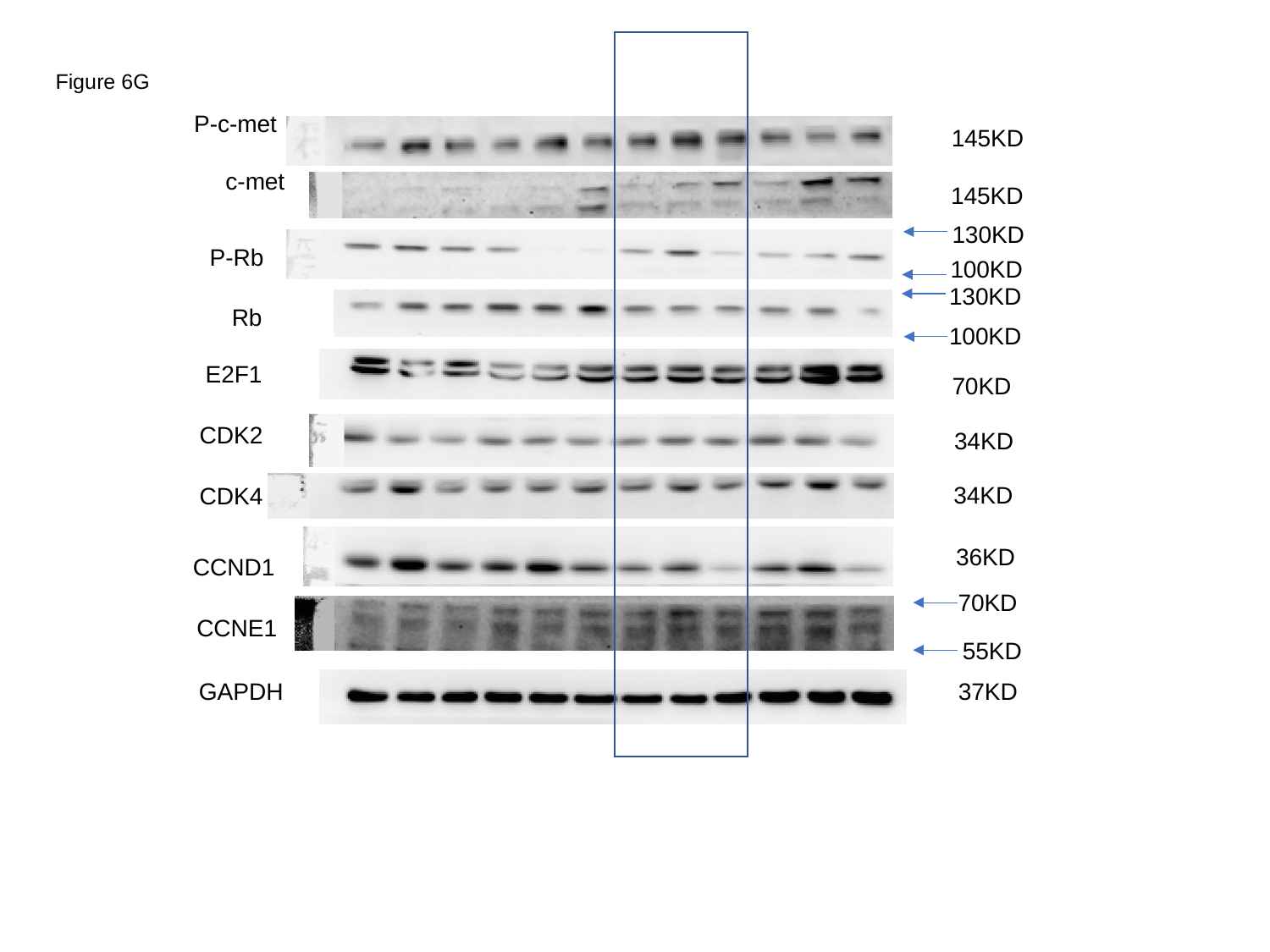

Figure 6G
P-c-met
145KD
c-met
145KD
130KD
P-Rb
100KD
130KD
Rb
100KD
E2F1
70KD
CDK2
34KD
34KD
CDK4
36KD
CCND1
70KD
CCNE1
55KD
GAPDH
37KD

## Slide 10
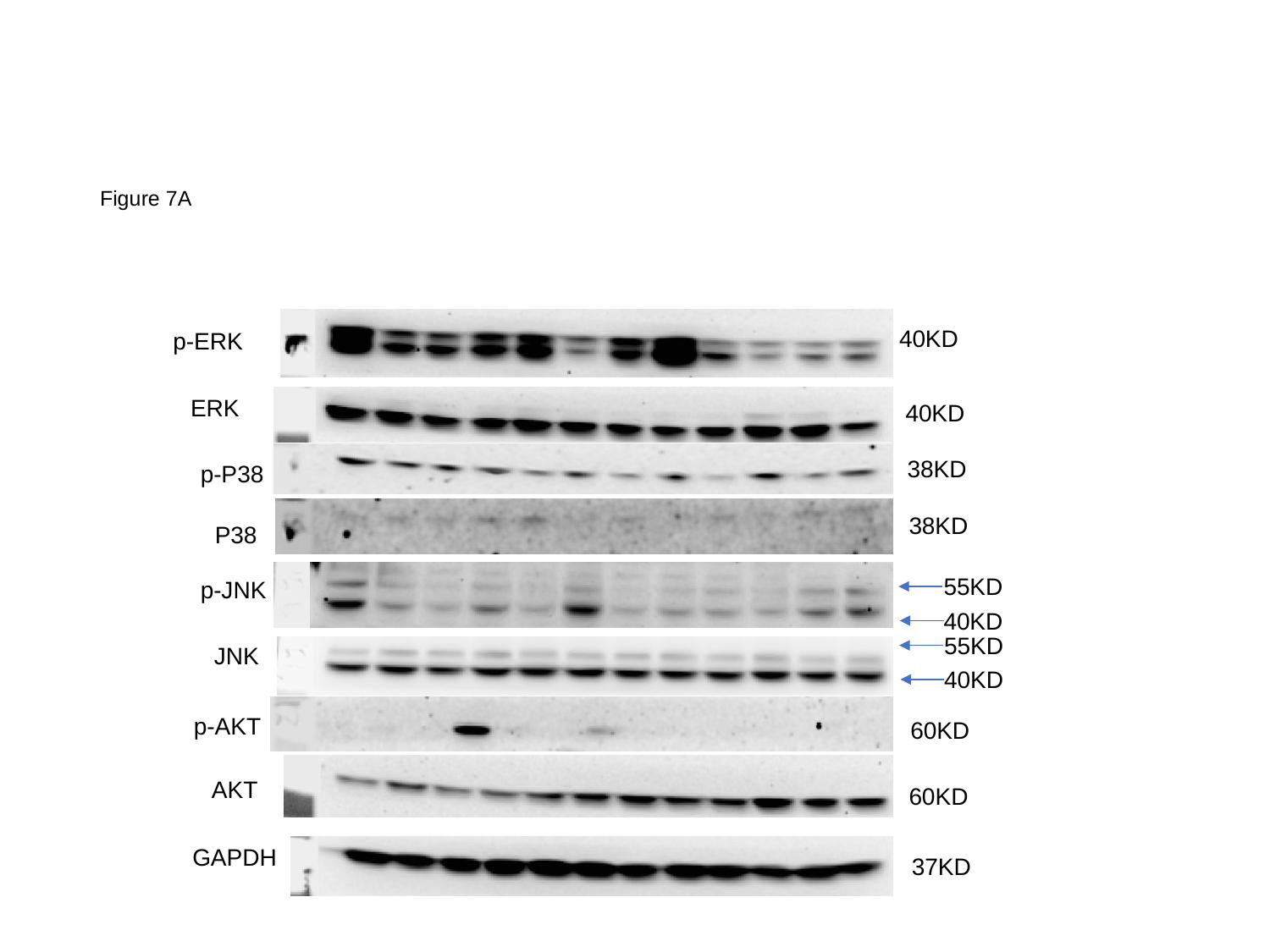

# Figure 7A
40KD
p-ERK
ERK
40KD
38KD
p-P38
38KD
P38
55KD
p-JNK
40KD
55KD
JNK
40KD
p-AKT
60KD
AKT
60KD
GAPDH
37KD

## Slide 11
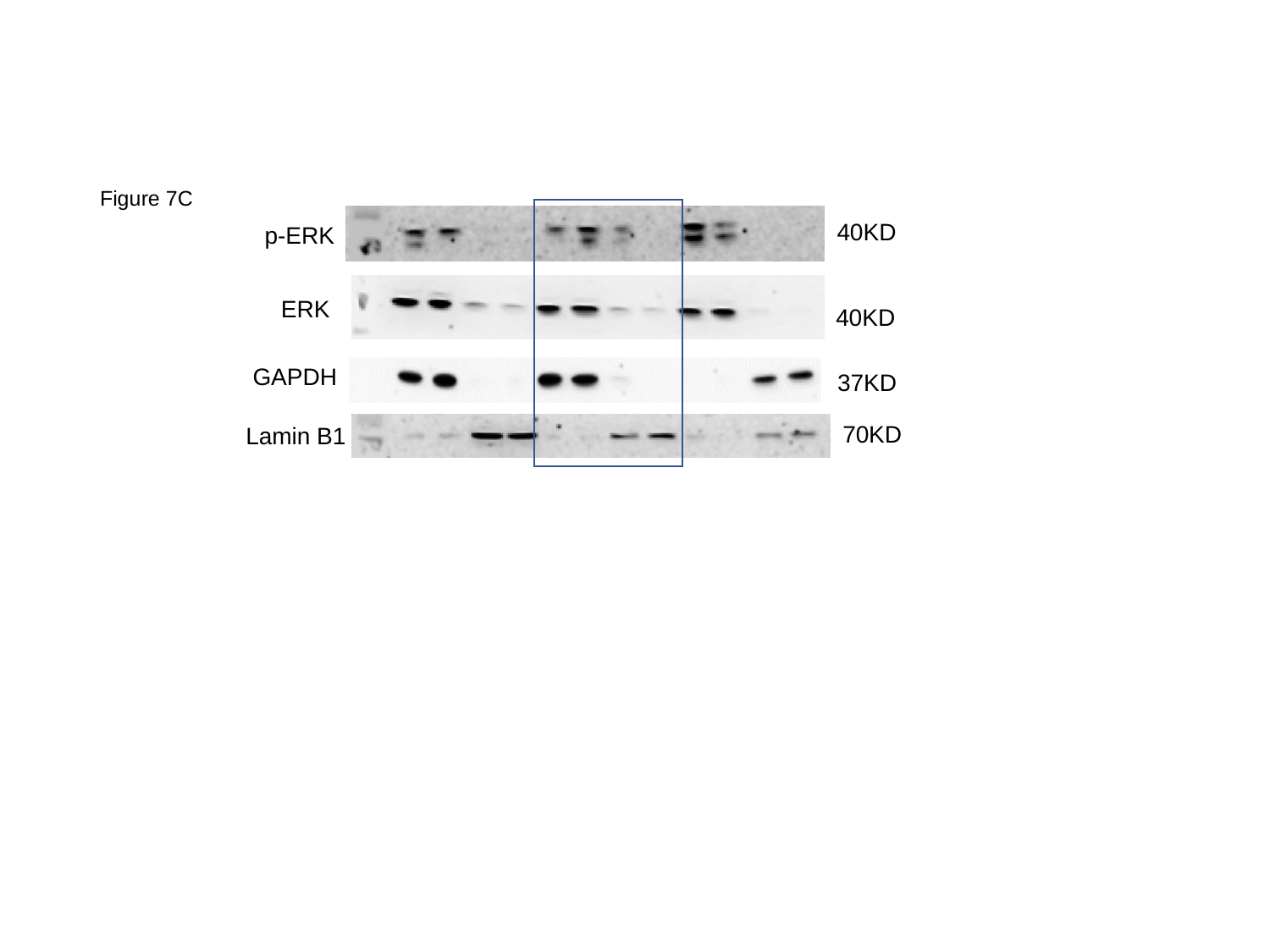

# Figure 7C
40KD
p-ERK
ERK
40KD
GAPDH
37KD
70KD
Lamin B1

## Slide 12
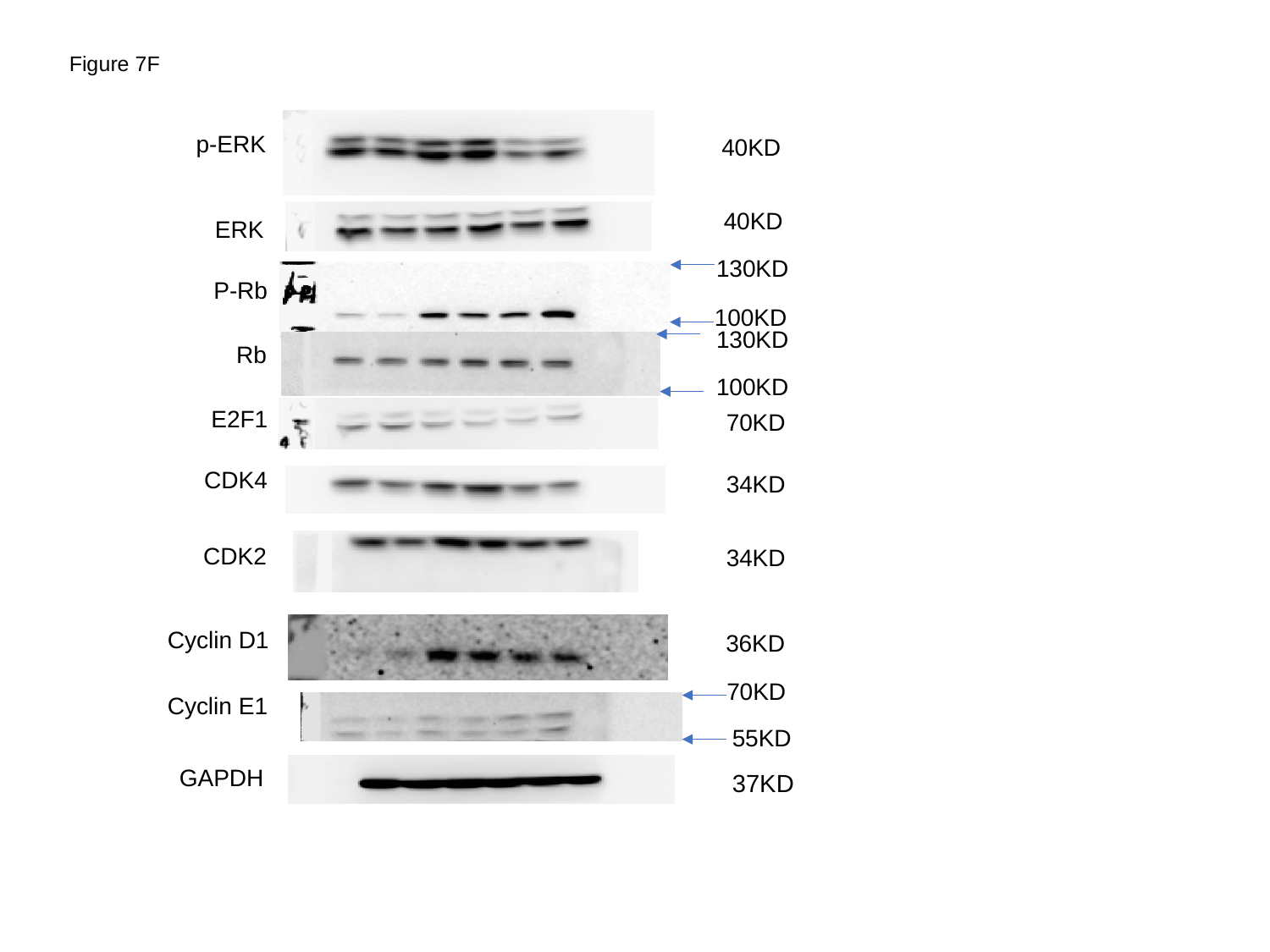

# Figure 7F
p-ERK
40KD
40KD
ERK
130KD
P-Rb
100KD
130KD
Rb
100KD
E2F1
70KD
CDK4
34KD
CDK2
34KD
Cyclin D1
36KD
70KD
Cyclin E1
55KD
GAPDH
37KD

## Slide 13
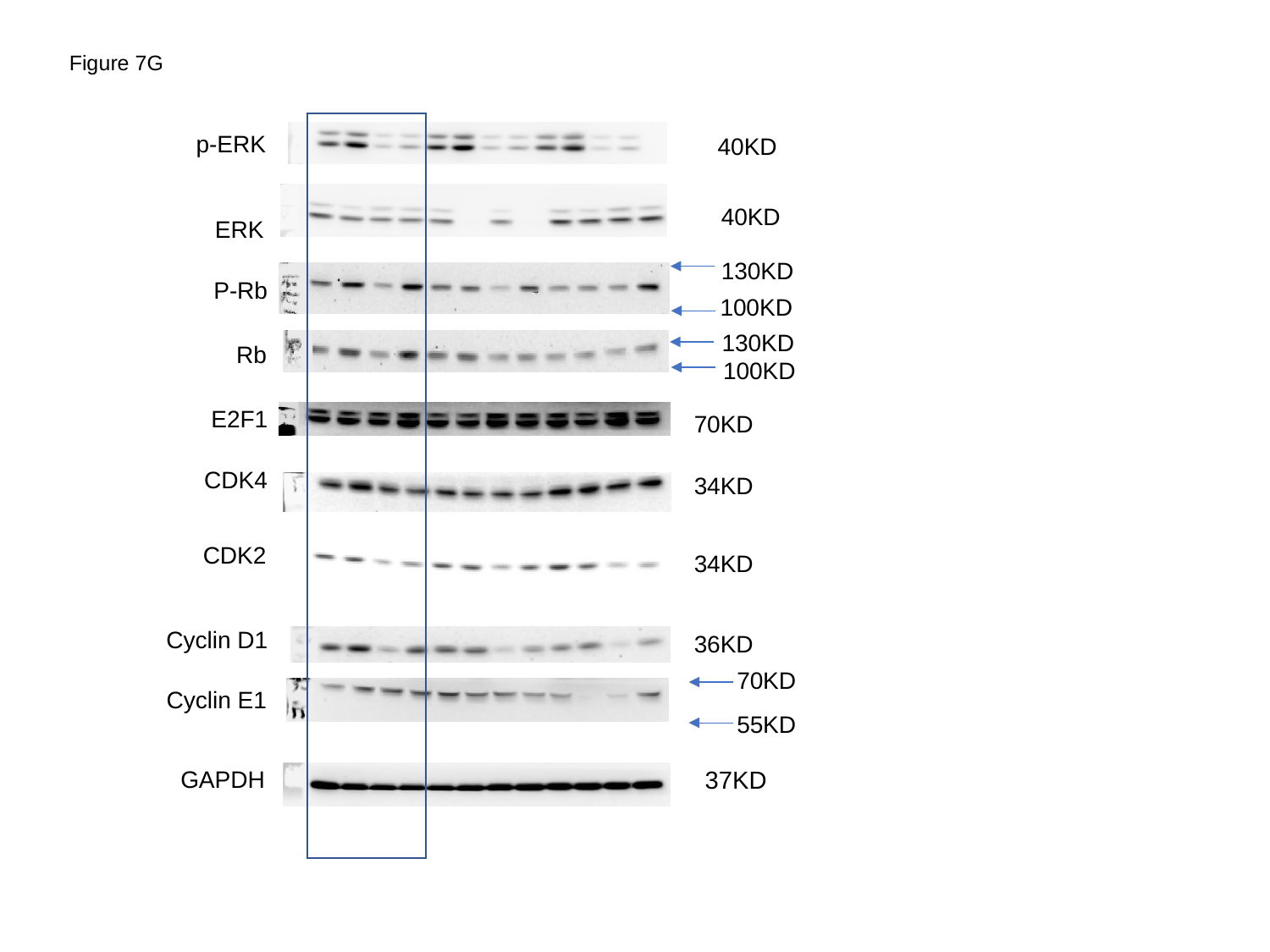

Figure 7G
p-ERK
40KD
40KD
ERK
130KD
P-Rb
100KD
130KD
Rb
100KD
E2F1
70KD
CDK4
34KD
CDK2
34KD
Cyclin D1
36KD
70KD
Cyclin E1
55KD
GAPDH
37KD

## Slide 14
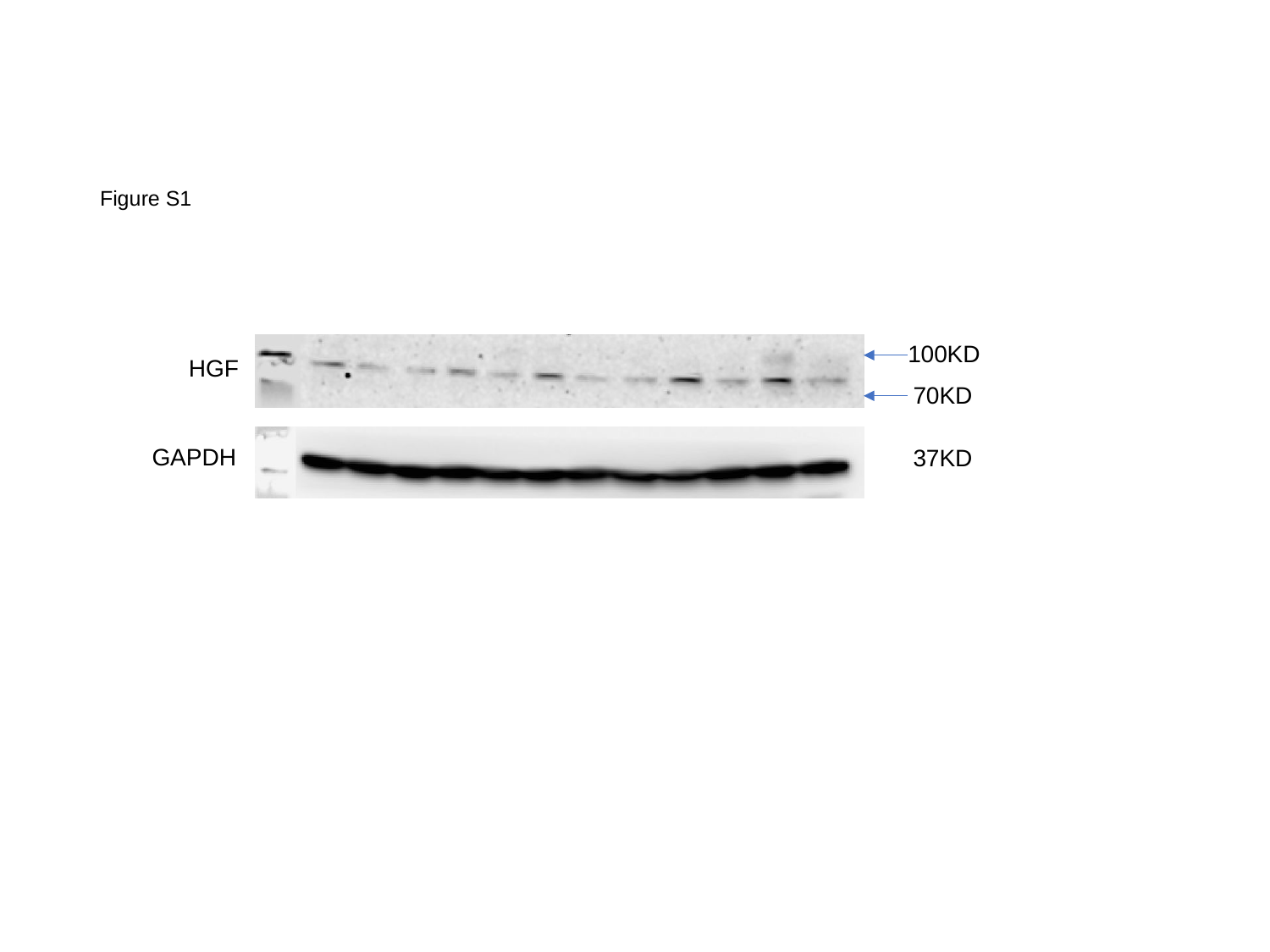

# Figure S1
100KD
HGF
70KD
GAPDH
37KD
